# Supplementary material for: Tbx5a lineage tracing shows cardiomyocyte plasticity during zebrafish heart regeneration
Source: Nat Commun. 2018 Jan 30;9:428. doi: 10.1038/s41467-017-02650-6 (PMC5789846; doi:10.1038/s41467-017-02650-6)
Supplement: Supplementary file 2 — Description of Additional Supplementary Files [file 41467_2017_2650_MOESM2_ESM.docx]

**Description of Additional Supplementary Files**

File Name: Supplementary Data 1

Description: List of genes differentially expressed between *tbx5a*^+^ and *tbx5a*^-^ cardiomyocytes. GFP^+^/nuc-dsRed^+^ and GFP^-^/nuc-dsRed^+^ cardiomyocytes were FAC sorted from adult *tbx5a:GFP;myl7:nuc-dsRed* cardiac ventricles.

File Name: Supplementary Data 2

Description: Sequence of the plasmid used for *tbx5a:tdTomato* BAC recombineering.

File Name: Supplementary Data 3

Description: Sequence of the plasmid containing *mCherry-p2a-CreER^T2^-flp-kan-flp* used as a template for recombineering.

File Name: Supplementary Data 4

Description: Sequence of the plasmid containing *iTol2Amp-Cryst:RFP* used as a template for recombineering.

File Name: Supplementary Data 5

Description: Sequence of the plasmid containing *loxP_tagBFP_loxP_mCherry-NTR- flp-kan-flp* used as a template for recombineering.

File Name: Supplementary Movie 1

Description: Confocal optical sections of a *tbx5a:GFP*;*myl7*:*mbmCherry* heart at 72 hpf. GFP (green) labels *tbx5a*^+^ cells and mCherry (red) marks cells expressing the panmyocardial marker *myosin light chain 7* (*myl7*). Shown are ventral views, cranial is to the top. *tbx5a*:GFP^-^ cardiomyocytes can be observed in the distal ventricle. at, atrium; v, ventricle. Scale bar 10 µm.

File Name: Supplementary Movie 2

Description: Confocal optical sections of a 56 hpf *tbx5a*:*GFP*;*drl*:*mCherry* double transgenic zebrafish heart. GFP (green) labels *tbx5a*^+^ cells, mCherry (red) *drl*^+^ cells and anti-myosin heavy chain (MHC) immunofluorescence all cardiomyocytes. at, atrium; v, ventricle. Scale bar 10 µm.

File Name: Supplementary Movie 3

Description: Confocal optical sections of 72 hpf *tbx5a*:*GFP*;*drl*:*mCherry* double transgenic zebrafish heart. GFP (green) labels *tbx5a*^+^ cells, mCherry (red) *drl*^+^ cells and anti-myosin heavy chain (MHC) immunofluorescence all cardiomyocytes. at, atrium; v, ventricle. Scale bar 10 µm.

File Name: Supplementary Movie 4

Description: Confocal optical sections of a heart at 72 hpf from a *tbx5a*:*GFP* lavae injected with an *ltbp3:mCherry* construct at the 1-cell stage. GFP labels *tbx5a*^+^ cells, mCherry *ltbp3*^+^ cells, and anti-myosin heavy chain (MHC) all cardiomyocytes.

File Name: Supplementary Movie 5

Description: Confocal optical sections of a *tbx5a*:*CreER^T2^*;*ubb:loxP-GFP-STOP-loxP-mCherry* zebrafish heart at 4 dpf, treated with 5 µM 4-OHT from 24 to 48 hpf. Recombined cells are shown in red and MHC in green. Note the completely recombined atria. at, atrium; v, ventricle. Scale bar 10 µm.

File Name: Supplementary Movie 6

Description: *tbx5a:CreER^T2^*; *vmhcl:loxP-tagBFP-loxP-mCherry-NTR* were treated with 4-OHT from 24 to 48 hpf, and then divided into a control group (-Mtz) and a group treated with Metronidazol (+Mtz) from 4 to 7 dpf. Shown are lateral views, the head is to the left. Larvae were imaged at 7 dpf. Note the differences in ventricular contractility between hearts from both groups.
