# Supplementary material for: Tbx5a lineage tracing shows cardiomyocyte plasticity during zebrafish heart regeneration
Source: Nat Commun. 2018 Jan 30;9:428. doi: 10.1038/s41467-017-02650-6 (PMC5789846; doi:10.1038/s41467-017-02650-6)
Supplement: Supplementary file 3 — Supplementary Information [file 41467_2017_2650_MOESM3_ESM.pdf]

Supplementary Figures 1-17 and Table 1

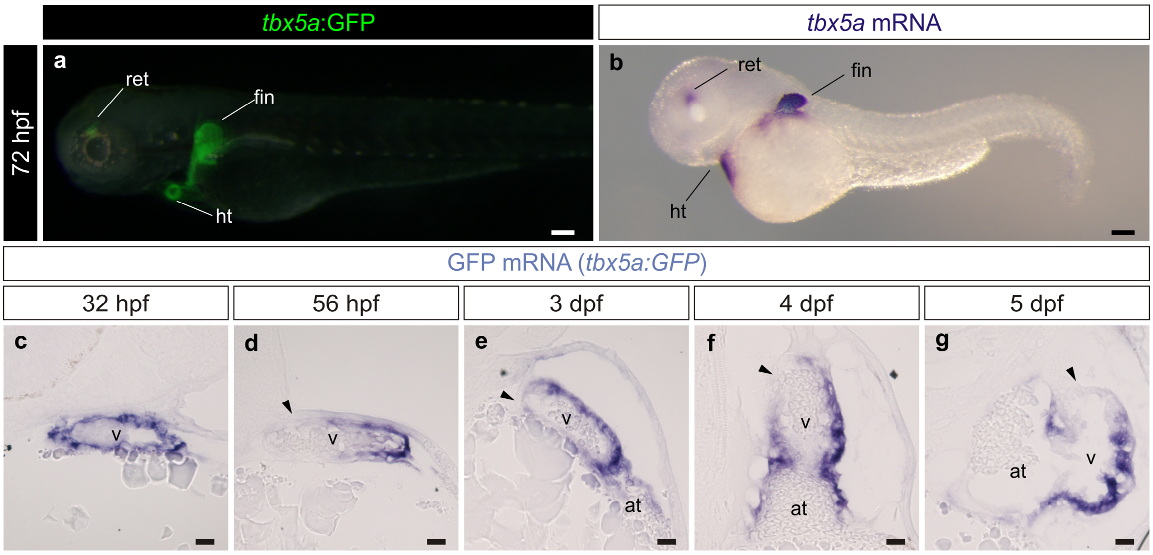

Supplementary Figure 1. Characterization of the *tbx5a*:GFP reporter line. a Lateral view of a *tbx5a*:*GFP* larvae at 3 days postfertilisation. A merged fluorescent and brightfield image is shown. **b** mRNA *in situ* hybridization (ISH) with a *tbx5a* antisense riboprobe on the same staged larvae as the one shown in **a** (n=7/7). **c**–**g** *gfp* mRNA on embryonic heart sections of *tbx5a:GFP* fish at different stages of development**.** The black arrowheads point to the *tbx5a*:GFP^{-} area of the ventricle. at, atrium; dpf, days postfertilisation; hpf, hours postfertilisation; ht, heart tube; ret, retina; v, ventricle. Scale bars, **a**, **b** 100 µm and **c**, **g** 25 µm

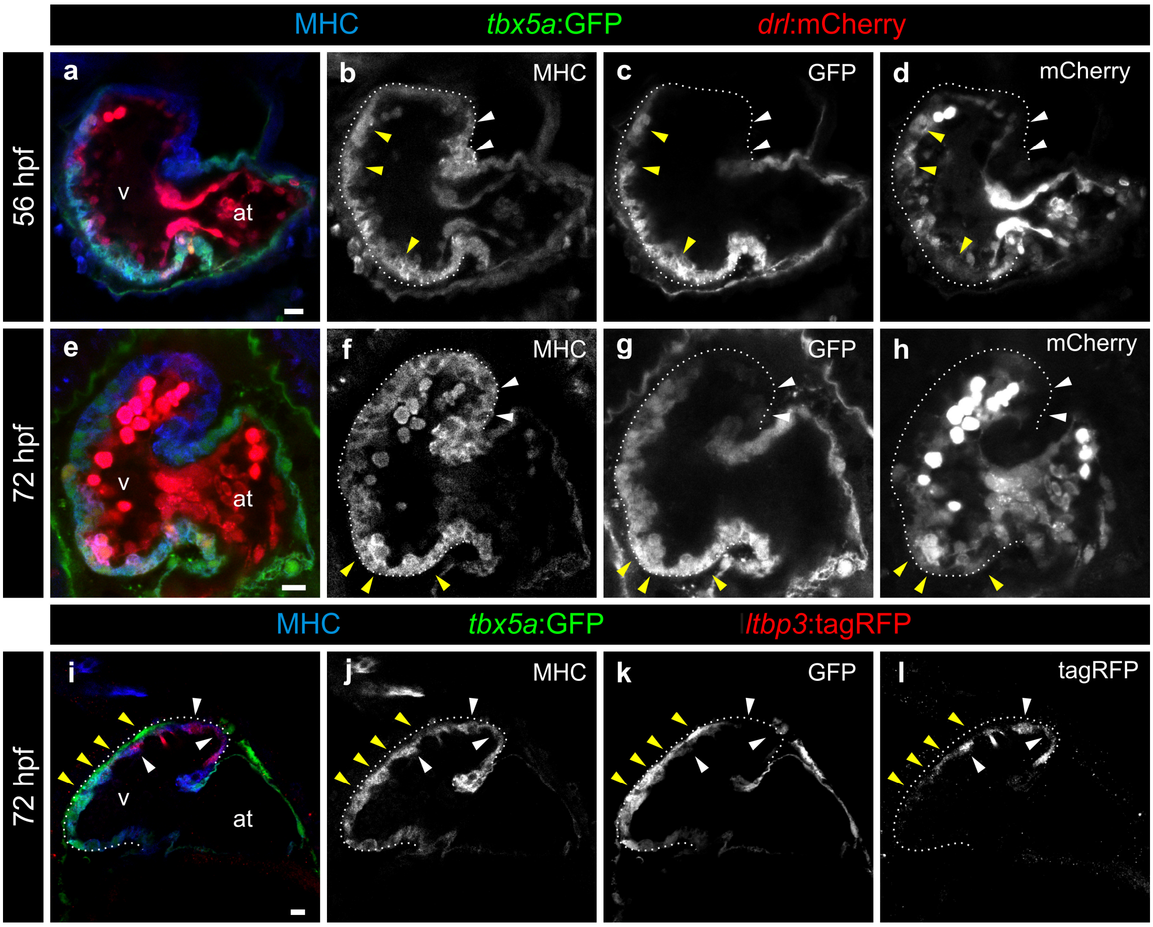

Supplementary Figure 2. The pattern of *tbx5a:*GFP expression is similar to that of *drl*:mCherry and complementary to *ltbp3*:mCherry. a–h Confocal optical sections of 56 (n=5/5) and 72 (n=7/7) hours postfertilisation (hpf) *tbx5a*:*GFP*;*drl*:*mCherry* double transgenic zebrafish larvae. GFP (green) labels *tbx5a*$^{+}$ cells, mCherry (red) *drl*$^{+}$ cells and anti-Myosin Heavy Chain (MHC) immunofluorescence labels all cardiomyocytes. The ventricle is outlined with dotted lines. The *tbx5a:*GFP^{-} and *drl:*mCherry^{-} distal ventricle is marked with white arrowheads, while the yellow arrowheads point to the domains positive for both markers. Note that *drl:*mCherry is also expressed in endocardial cells and red blood cells in the lumen of the heart. **i–l** Confocal optical sections of 72 hpf hearts from *tbx5a*:*GFP* embryos injected with *ltbp3:TagRFP-2A-Cre* at the one cell stage. Shown is a representative heart out of 8 larvae. GFP labels *tbx5a*$^{+}$ cells, mCherry *ltbp3*$^{+}$ cells, and MHC all cardiomyocytes. The yellow arrowheads point to *tbx5a:*GFP^{+} cells that are *ltbp3:*mCherry^{-} while the white arrowhead points to a *ltbp3*:mCherry^{+} cells within the *tbx5a:*GFP^{-} domain. at, atrium; v, ventricle. Scale bars, 10 µm

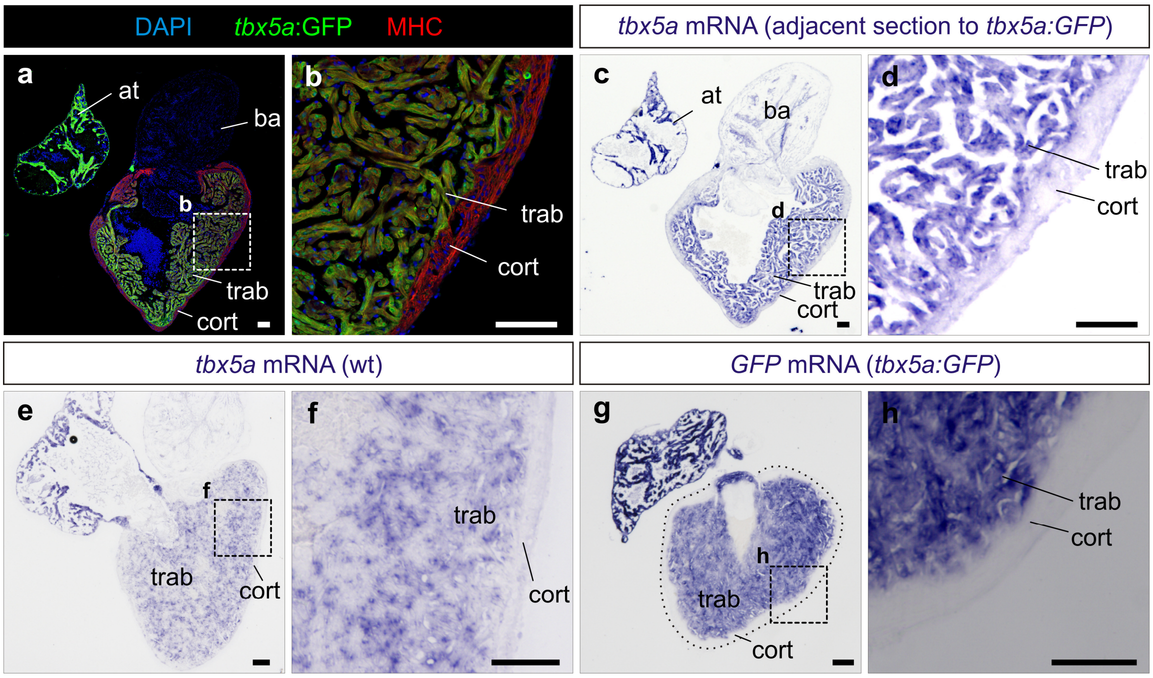

Supplementary Figure 3. The transgenic *tbx5a:GFP* reporter line recapitulates the expression of endogenous *tbx5a*. **a**,**b** Sagittal section through an adult *tbx5a*:*GFP* zebrafish heart immunostained for GFP and Myosin Heavy Chain (MHC), (n=8/8). **c**,**d** mRNA *in situ* hybridization (ISH) with a *tbx5a* antisense riboprobe on the adjacent section shown in **a** and **b**. Note expression of GFP and *tbx5a* mRNA in the trabecular myocardium and their absence of expression in the cortical myocardium (n=8/8). **b** and **d** are zoomed views of boxed areas in **a** and **c**. **e**,**f** *tbx5a* ISH on a sagittal heart section of an AB wildtype adult zebrafish (n=5). **g**,**h** *GFP* ISH on a sagittal heart section of *tbx5a*:*GFP* zebrafish (n=4). at, atrium; ba, bulbus arteriosus; cort, cortical layer; trab, trabecular layer; wt, wildtype. Scale bars, 100 µm

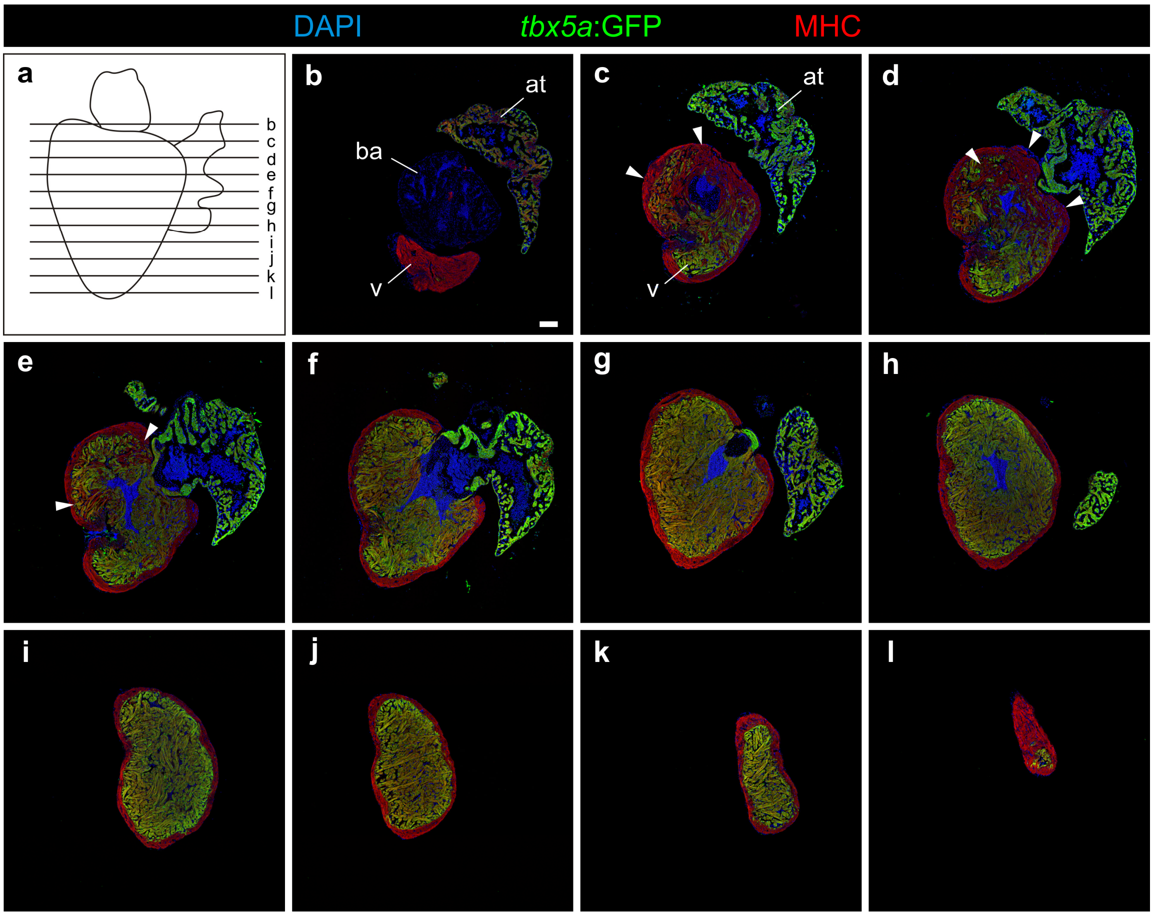

Supplementary Figure 4. Serial sections of a *tbx5a*:*GFP* transgenic adult heart.

a Scheme showing the direction used for sectioning the heart and domain represented in images **b**–**l**. **b**–**l** Serial sections of a *tbx5a*:*GFP* transgenic adult heart immunostained for GFP (*tbx5a*$^{+}$ cells, green) and Myosin Heavy Chain (MHC) (cardiomyocytes, red). Nuclei are counterstained with DAPI. Arrowhead marks the *tbx5a*:GFP negative region in the basal ventricle (n=13/13). at, atrium; ba, bulbus arteriosus; v, ventricle. Scale bar, 100 µm

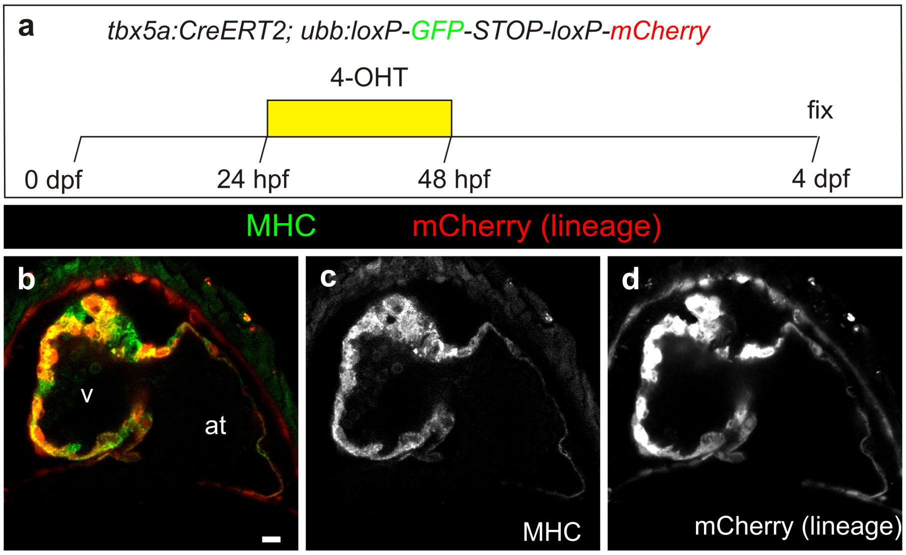

Supplementary Figure 5. Fate mapping of embryonic *tbx5a*-derived cells.
a–d Optical section through a *tbx5a*:*CreER*^{T2};*ubb*:*loxP-GFP-loxP-mCherry* heart at 4 days postfertilisation (dpf) treated with 4-Hydroxytamoxifen (4-OHT) as shown in **a** revealing efficient recombination in ventricular and atrial cardiomyocytes (mCherry, red). Shown is a section through the atrioventricular canal region. The distal *tbx5a*$^{-}$ region is not visible in this section. See Supplementary Movie 5 for a full z-stack visualization. at, atrium; hpf, hours postfertilisation; v, ventricle. Scale bars, 10 µm

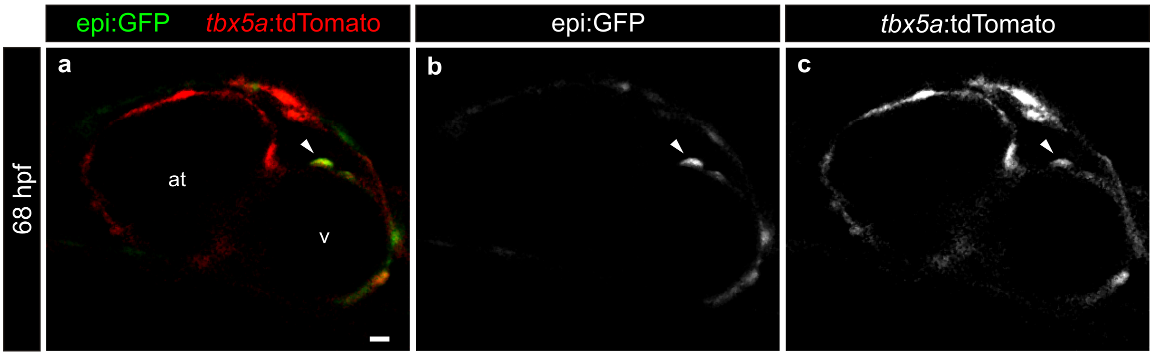

Supplementary Figure 6. *tbx5a*:tdTomato colocalizes with an epicardial marker transgenic line. Shown is an optical section of a heart at 68 hours postfertilisation (hpf) from a double transgenic *tbx5a*:tdTomato^{cn13}; *Et(-26.5Hsa.WT1-gata2:EGFP)*^{cn12} animal. Arrowhead marks a double positive epicardial cells on the ventricle (observed in 6 out of 7 larvae). at, atrium; v, ventricle. Scale bar, 10 µm

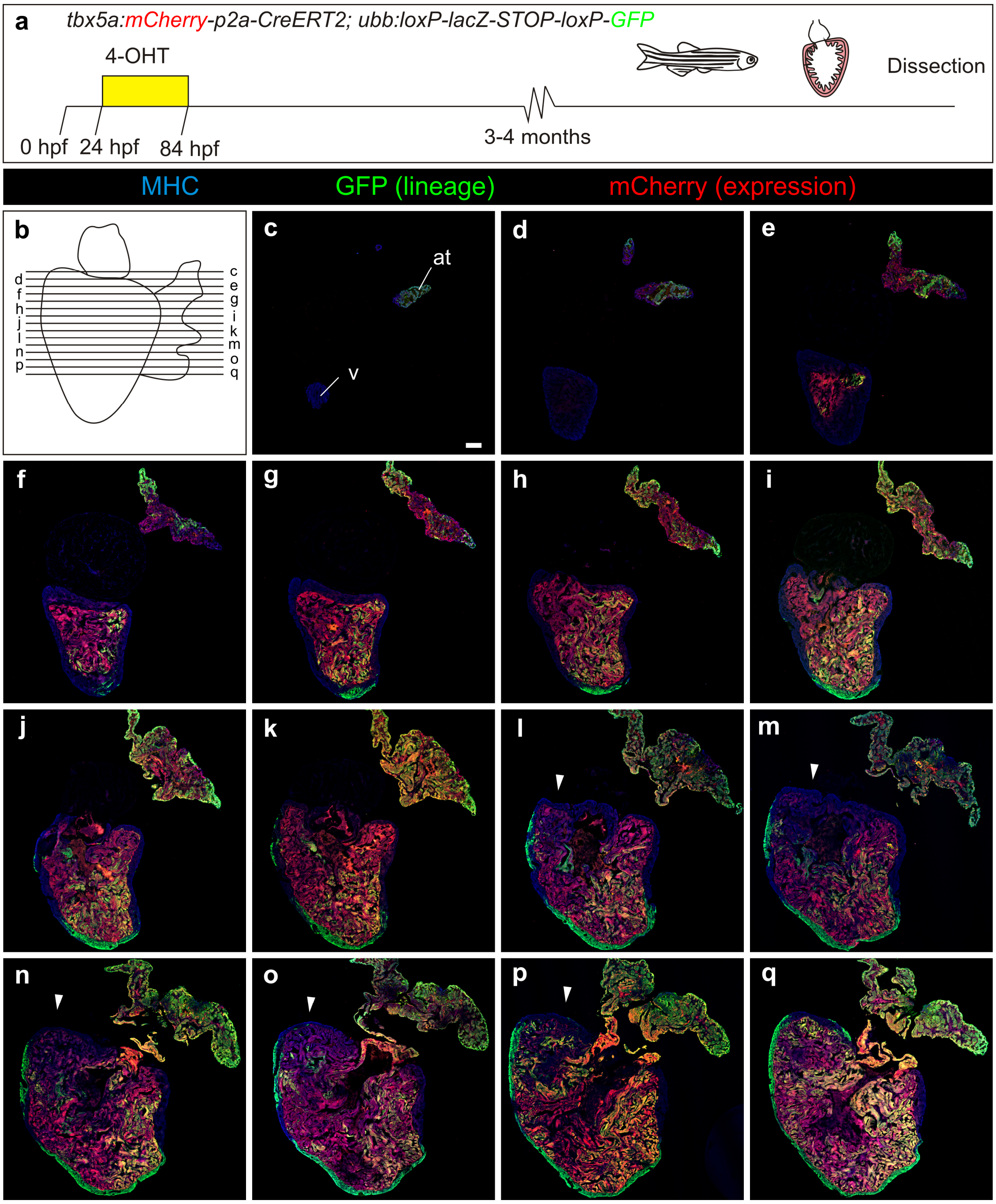

Supplementary Figure 7. Fate mapping of embryonic *tbx5a*-derived cells on transverse sections of adult hearts.

a Overview of the experimental setup. **b** Scheme showing the sectioning orientation through the heart and the location of the individual sections shown in the figure. **c–q** Immunofluorescence staining of adult heart sections recombined as in **a**. Shown are merged channels for GFP (green), mCherry (red) and anti-MHC staining (blue). Arrowheads point to the negative basal domain in the ventricle (n=2/2). at, atrium; v, ventricle. Scale bar, 100 µm

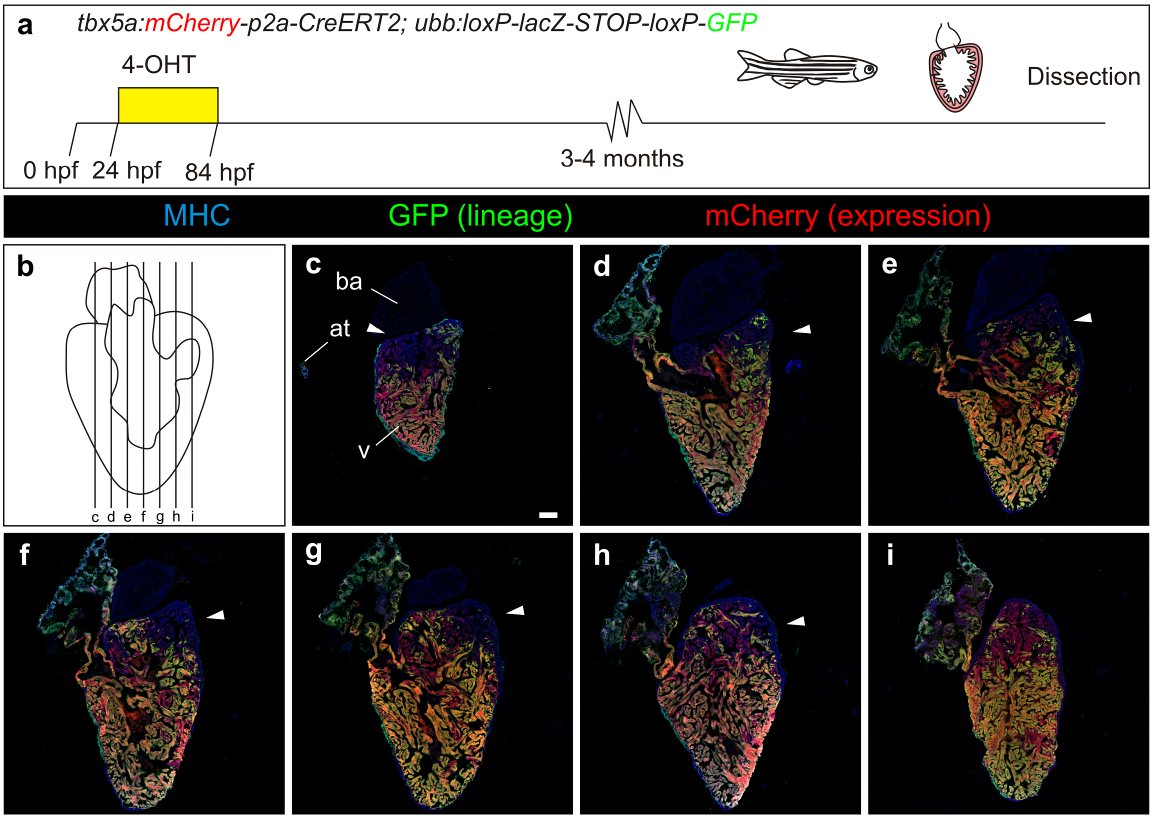

Supplementary Figure 8. Fate mapping of embryonic *tbx5a*-derived cells on sagittal sections of adult hearts.

a Overview of the experimental setup. **b** Scheme showing the sectioning orientation through the heart and the location of the individual sections shown in the figure. **c**–**i** Immunofluorescence staining of adult heart sections recombined as in **a**. Shown are merged channels for GFP (green), mCherry (red) and anti-MHC staining (blue). Arrowheads point to the negative basal domain in the ventricle n=3/3. Scale bar, 100 µm

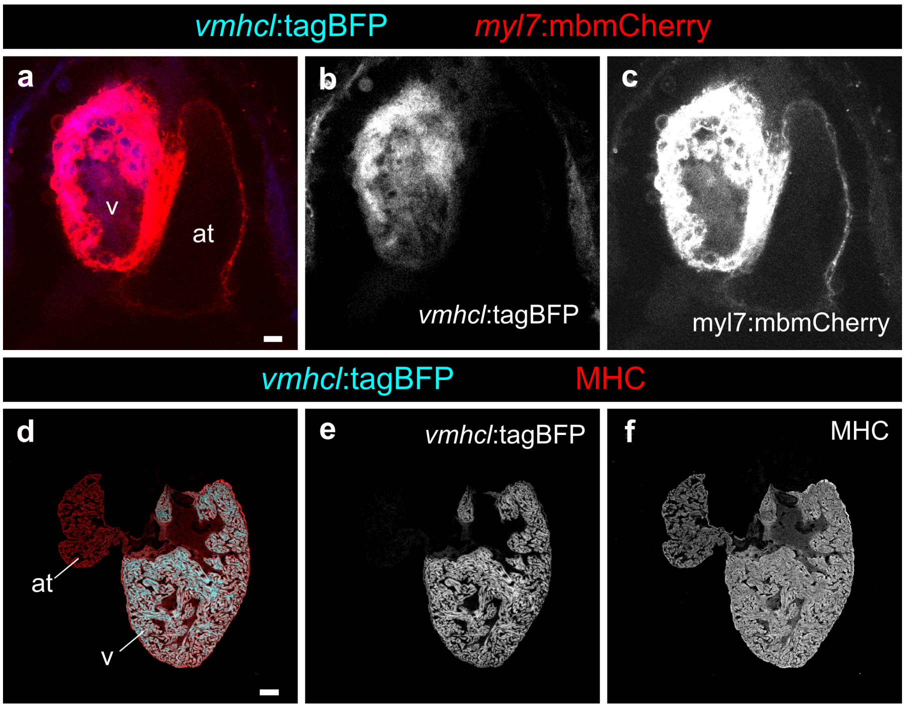

Supplementary Figure 9. Characterization of the *vmhcl* promoter to drive expression specifically in the cardiac ventricle.

a–**c** Heart from larvae at 4 days postfertilisation (dpf) showing that the *vmhcl:loxP-tagBFP-loxP-mCherry-NTR* line is specific for ventricular cardiomyocytes (n=10/10). **d**–**f** Specificity and expression is maintained in the adult (n=9/9). at, atrium; v, ventricle. Scale bars, **a** 10 µm, **d** 100 µm

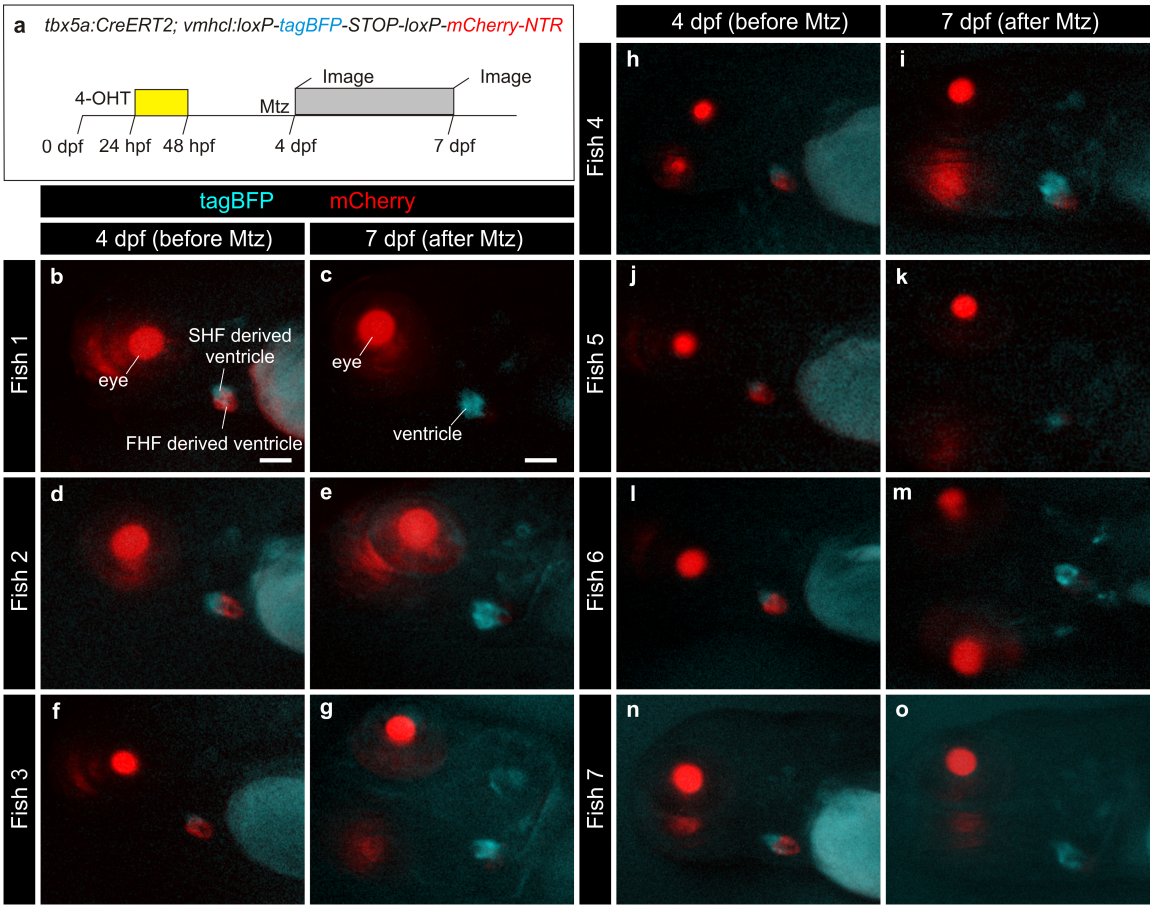

Supplementary Figure 10. Assessment of efficient ablation of *tbx5a*-derived cardiomyocytes in individualized fish. a *tbx5a*$^{+}$ ventricular cardiomyocytes were genetically ablated in *tbx5a:CreER*^{T2}*;vmhcl:loxP-tagBFP-loxP-mCherry-NTR* double transgenic zebrafish by administration of Metronidazole (Mtz). Recombination was induced by administration of 4-Hydroxytamoxifen (4-OHT) at 1 and 2 days postfertilisation (dpf). At 4 dpf, larvae where imaged under a binocular scope. **b–o** Images show lateral views of the head and heart region. The lens is red (gamma-crystallin transgenic reporter), the cardiac ventricle is red (mCherry^{+} *tbx5a*-derived ventricular cardiomyocytes) and blue (*tbx5a*$^{-}$ cardiomyocytes). Fish were individually treated with Mtz from 4 to 7 dpf. At 7 dpf, 3 days after initiation of Mtz treatment, each larva was imaged again. At this stage, tagBFP expression is visible and expanded but mCherry is no longer detected in most larvae. FHF, first heart field; SHF, second heart field. Scale bars, 100 µm

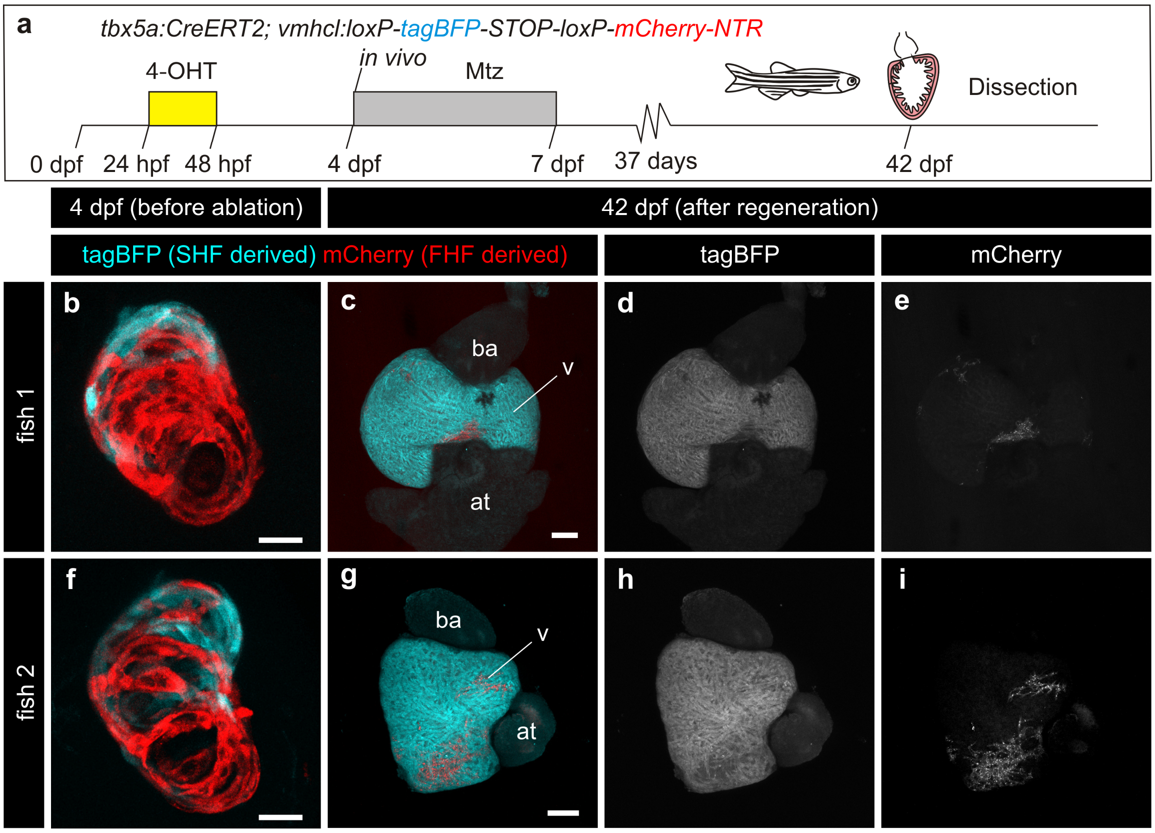

Supplementary Figure 11. Genetic ablation of *tbx5a*-derived ventricular cardiomyocytes in individualized fish. a *tbx5a*$^{+}$ ventricular cardiomyocytes were genetically ablated in *tbx5a:CreER*^{T2}*;vmhcl:loxP-tagBFP-loxP-mCherry-NTR* double transgenic zebrafish. Recombination was induced by administration of 4-Hydroxytamoxifen (4-OHT). Fish were individualised, imaged at 4 days postfertilisation (dpf) and cell ablation was induced by administration of Metronidazole (Mtz) from 4 to 7 dpf. **b**,**f** Maximum projection of confocal z-stacks of 4 dpf embryos. **c**–**e**, **g**–**i** Hearts dissected from the same fish at 42 dpf. at, atrium; ba, bulbus arteriosus; v, ventricle. Scale bars, **b**, **f** 25 µm, **c**, **g** 100 µm

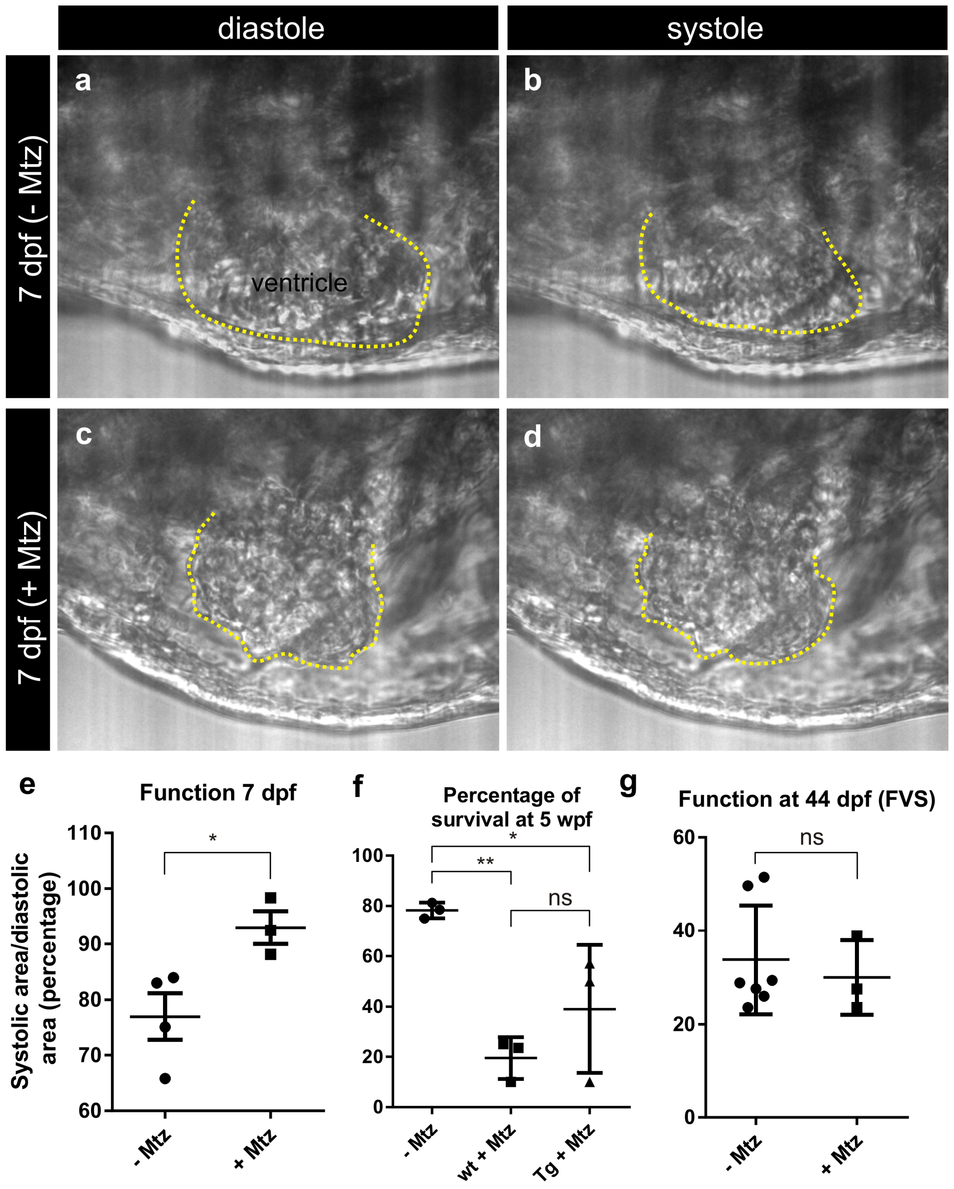

Supplementary Figure 12. Cardiac performance after ablation of *tbx5a*-derived cells. *tbx5a*$^{+}$ ventricular cardiomyocytes were genetically ablated in *tbx5a:CreER*^{T2};*vmhcl:loxP-tagBFP-loxP-mCherry-NTR* double transgenic zebrafish. Recombination was induced by administration of 4-Hydroxytamoxifen (4-OHT) at 1 and 2 days postfertilisation. Animals were divided into two groups. Cell ablation was induced in one group by administration of Metronidazole (Mtz) from 4 to 7 dpf. Videos were acquired at 7 dpf, immediately after the last Mtz treatment. **a**–**d** are still images from the videos from two Mtz-treated (from a total of 4) and two non-treated (from a total of 3) animals. Shown are lateral views of the heart, the head is to the left. The ventricle is outlined in yellow. Note the irregular shape and overall smaller area in Mtz-treated hearts. **e** The maximum (diastolic) and minimum (systolic) ventricular area was measured to determine ventricular function. P= 0.0348 by a

two-tailed t-test. Deficient contraction was detected upon Mtz treatment. **f** Percentage of survival at 5 weeks post fertilization (wpf); mean±s.d **P<0.01; *P<0.05; ns, non-significant by one-way ANOVA followed by Tukey's multiple comparisons test. Each point represents the survival percentage of a group comprising 14-22 fish each.
–Mtz: same stages control fish; sib+Mtz= *tbx5a*:*CreER*^{T2} and *vmhcl*:*loxP-tagBFP-loxP-mCherry-NTR* single transgenic siblings; Tg+Mtz= *tbx5a*:*CreER*^{T2};*vmhcl*:*loxP-tagBFP-loxP-mCherry-NTR* double transgenic fish. **g** Assessment of ventricular Fractional Volume Shortening (FVS) by echocardiography at 44 dpf. Shown are individual measurements as well as mean±s.d; ns, P = 0.6262 by two-tailed unpaired t-test; n=7 for – Mtz group and n=3 for + Mtz group

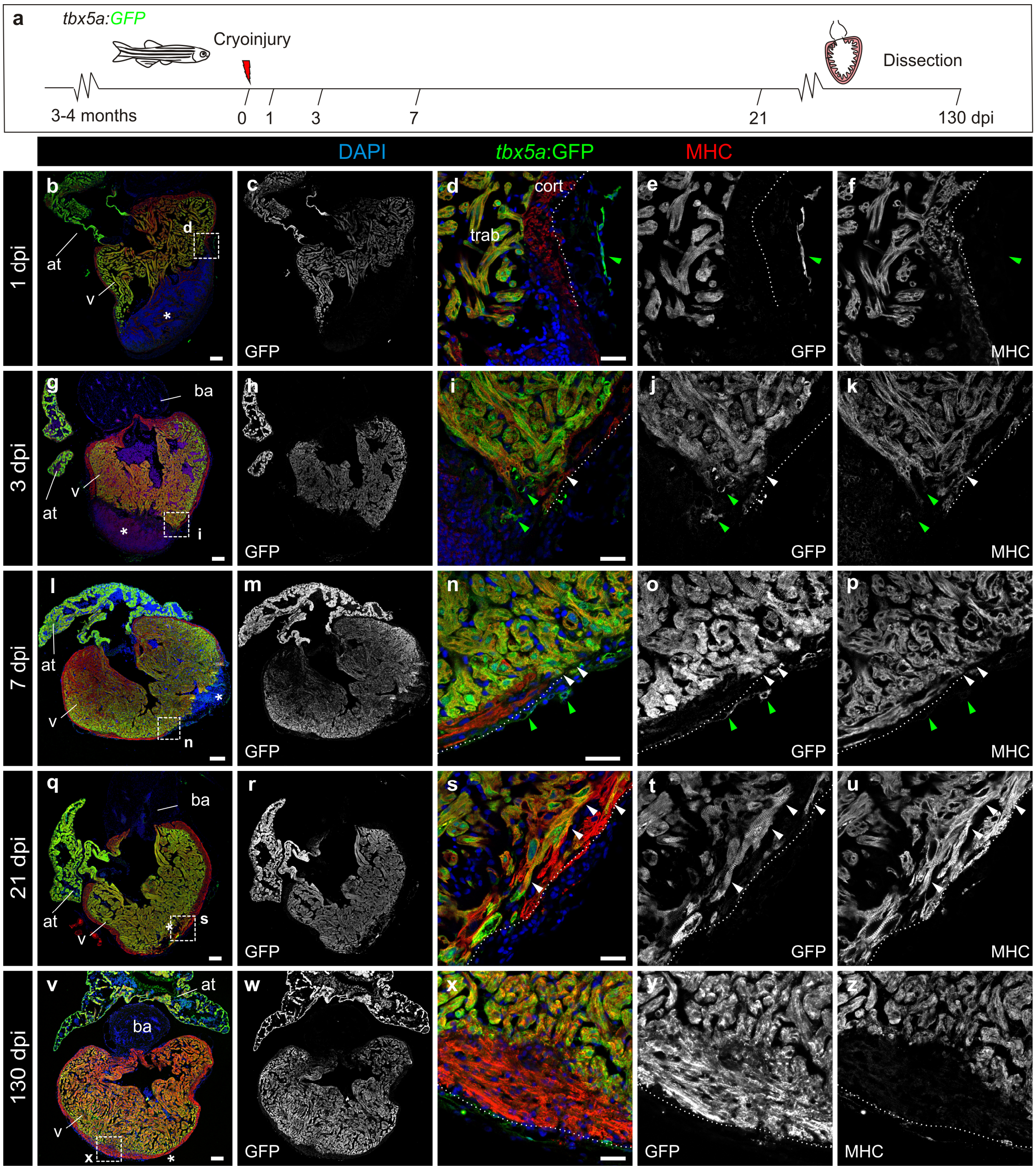

Supplementary Figure 13. Expression of *tbx5a*:GFP during adult heart regeneration. **a**, Illustration of the experimental setup. **b**–**z** Sagittal sections through *tbx5a:GFP* hearts at 1 (**b**–**f** n=4/4), 3 (**g**–**k** n=4/5), 7 (**l**–**p** n=3/3), 21 (**q**–**u** n=4/4) and 130 (**v**–**z** n=4/4) days post injury (dpi). Sections were immunostained with anti-GFP (green) and anti-Myosin Heavy Chain (MHC; red). Nuclei are counterstained with DAPI (blue). Asterisks indicates the injury area, dotted line marks the outer border of the cortical layer. *tbx5a*:GFP expression is limited to the trabecular myocardium. Few myosin-negative *tbx5a*:GFP$^+$ cells are found within the epicardial layer (arrowhead). At 130 dpi, a *tbx5a*:GFP$^-$ thickened cortical myocardium covers a *tbx5a*:GFP$^+$ trabecular myocardium at the injury site (**v**–**z**). White arrowheads point to *tbx5a*:GFP$^+$ cardiomyocytes; green arrowheads label *tbx5a*:GFP$^+$ non-cardiomyocytes. Scale bars, **b**, **g**, **l**, **q**, **v** 100 µm, **d**, **i**, **n**, **s**, **x** 25 µm

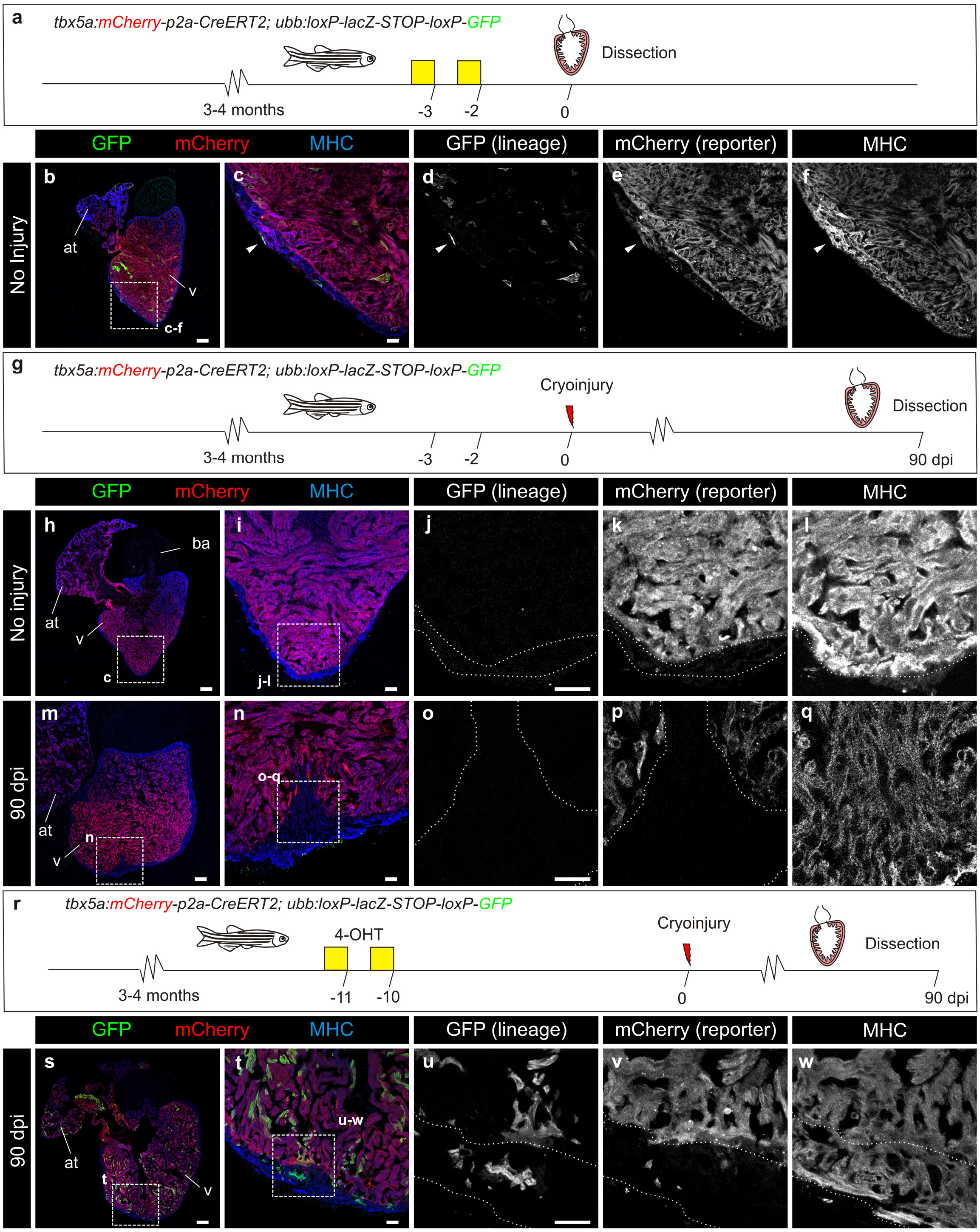

Supplementary Figure 14. Trabecular cardiomyocyte lineage tracing control experiments. a–f *tbx5a*:*mCherryp-2a-CreER*^{T2} was crossed into *ubb*:*loxP-lacZ-STOP-loxP-GFP*. Fish were treated with 4-Hydroxytamoxifen (4-OHT) at days 3 and 2 before fixation. Arrowhead points to one of the 2 cells GFP^{+} cells identified in 1 out of 6 hearts. **g–q** *loxP* sites in *tbx5a*:*Cherryp-p2a-CreER*^{T2};*ubb*:*loxP-lacZ-STOP-loxP-GFP* do not

recombine in the absence of 4-OHT. **g** *tbx5a:Cherry-p2A-CreER^{T2}* was crossed into *ubb*:*loxP-lacZ-STOP-loxP-GFP*. Fish were cryoinjured but not treated with 4-OHT. **h–l** Heart before injury. No recombined cells were observed (n = 4/4). **m–q** Hearts were injured and dissected at 90 days postinjury (dpi). No GFP^{+} recombined cells were visible at the regenerated area (n= 3/3). **r–w** Recombination is not due to the persistence of 4-OHT in the adult fish beyond cryoinjury. **r** *tbx5a*:*mCherry-p2a-CreER^{T2}* was crossed into *ubb*:*loxP-lacZ-STOP-loxP-GFP*. Fish were treated with 4-OHT during days 11 to 10 before the injury. **s–w** Contribution of the GFP^{+} trabeculae to the new compact layer and *tbx5a*:mCherry switch off in these cells can be observed (n=3/3). at, atrium; v, ventricle. Scale bars, **b**, **h**, **m** 100 µm, **c**, **i**, **j**, **n**, **o**, **t**, **u** 25 µm

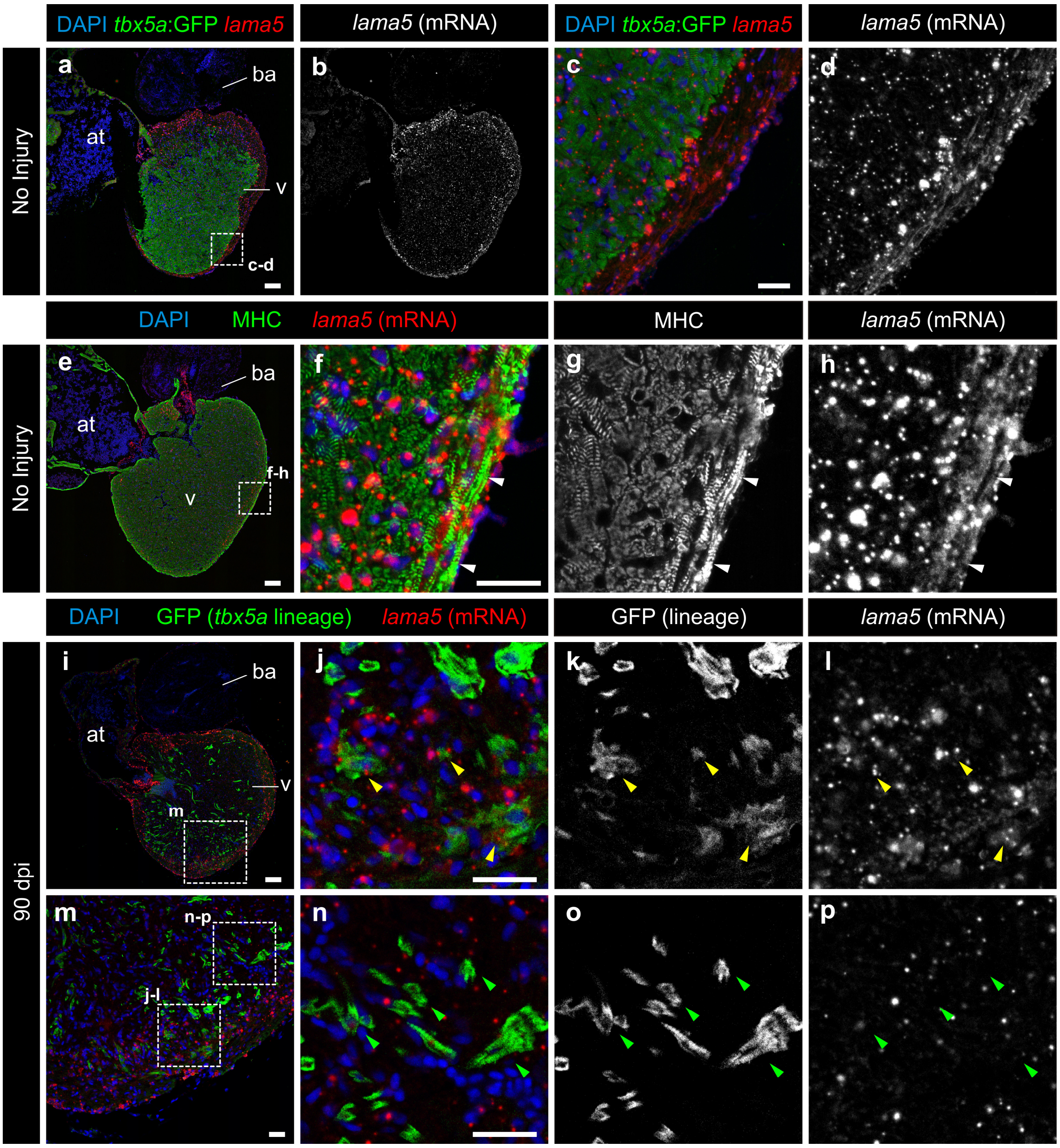

Supplementary Figure 15. *tbx5a*-derived cardiomyocytes within the cortical layer are *lama5* positive. *lama5* RNAScope *in situ* detection followed by GFP and Myosin Heavy Chain (MHC) immunofluorescence on sagittal sections of *tbx5a*:*GFP* (**a**–**h**) or *tbx5a*:*mCherryp-2a-CreER*^{T2};*ubb*:*loxP-lacZ-loxP-GFP* double transgenic animals, recombined before injury and fixed at 90 days postinjury (dpi) (**i**-**p**). Nuclei are counterstained with DAPI. **a**–**d** Uninjured heart. *tbx5a*:GFP (green) does not co-localize with *lama5* (red). *lama5* is expressed at higher and more homogenous levels in the cortical layer (n=4/4). **e**–**h** Uninjured heart. anti-MHC marks cardiomyocytes (green). In the cortical layer, regions of *lama5*/MHC co-localization were detected (white arrowheads)

(n=4/4). **i–p** 90 dpi regenerated hearts. *tbx5a*:*mCherryp-2a-CreER*^{T2};*ubb*:*loxP-lacZ-loxP-GFP* were treated with two 12 hours pulses of 4-Hydroxytamoxifen (4-OHT) at 6 and 7 days before the injury. GFP^{+} cells marking the *tbx5a* lineage within the cortical layer were positive for *lama5* (yellow arrowheads); double positive cells were observed in 11 out of 11 hearts. GFP^{+} cells within the trabecular layer were negative for that marker (green arrowheads; n=11/11). at, atrium; ba, bulbus arteriosus; v, ventricle. Scale bars, **a**, **e**, **i** 100 µm, **c**, **f**, **j**, **n** 25 µm

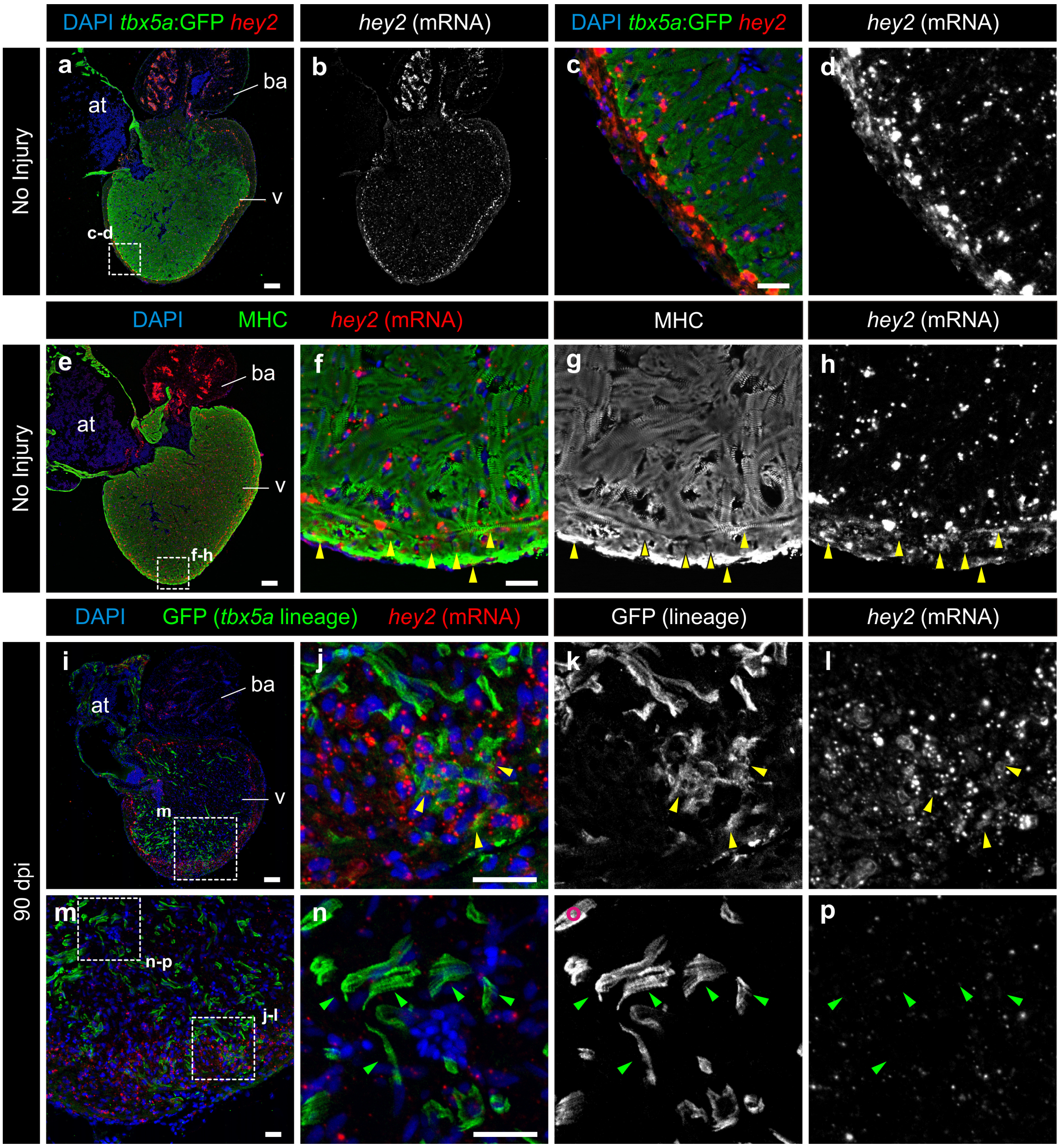

Supplementary Figure 16. *tbx5a*-derived cardiomyocytes within the cortical layer are *hey2* positive. GFP immunofluorescence and *hey2* RNAScope *in situ* detection on sagittal sections of *tbx5a*:*GFP* (**a**–**h**) or *tbx5a*:*mCherryp-2a-CreER*^{T2};*ubb*:*loxP-lacZ-loxP-GFP* double transgenic animals, recombined before injury and fixed at 90 days postinjury (dpi) (**i**–**p**). Nuclei are counterstained with DAPI. **a**–**d** Uninjured heart. *tbx5a*:GFP (green) does not co-localize with *hey2* (red). *Hey2* is expressed at higher and more homogenous levels in the cortical layer. **e**–**h** Uninjured heart. anti-Myosin Heavy Chain (MHC) marks cardiomyocytes (green). In the cortical layer, regions of *hey2*/MHC co-localisation were detected (yellow arrowheads; n=3/4). **i**–**p** 90 dpi regenerated hearts. *tbx5a*:*mCherryp-2a-*

CreER^{T2};*ubb*:*loxP-lacZ-loxP-GFP* were treated with two 12 hours pulses of 4-OHT at 6 and 7 days before the injury. GFP^{+} cells marking the *tbx5a* lineage within the cortical layer were positive for *hey2* (yellow arrowheads, *hey2*$^{+}$/GFP^{+} cells observed in 5 out of 6 hearts. GFP^{+} cells within the trabecular layer were negative for *hey2* (green arrowheads; n=6/6). at, atrium; ba, bulbus arteriosus; v, ventricle. Scale bars, **a**, **e**, **i** 100 µm, **c**, **f**, **j**, **n** 25 µm

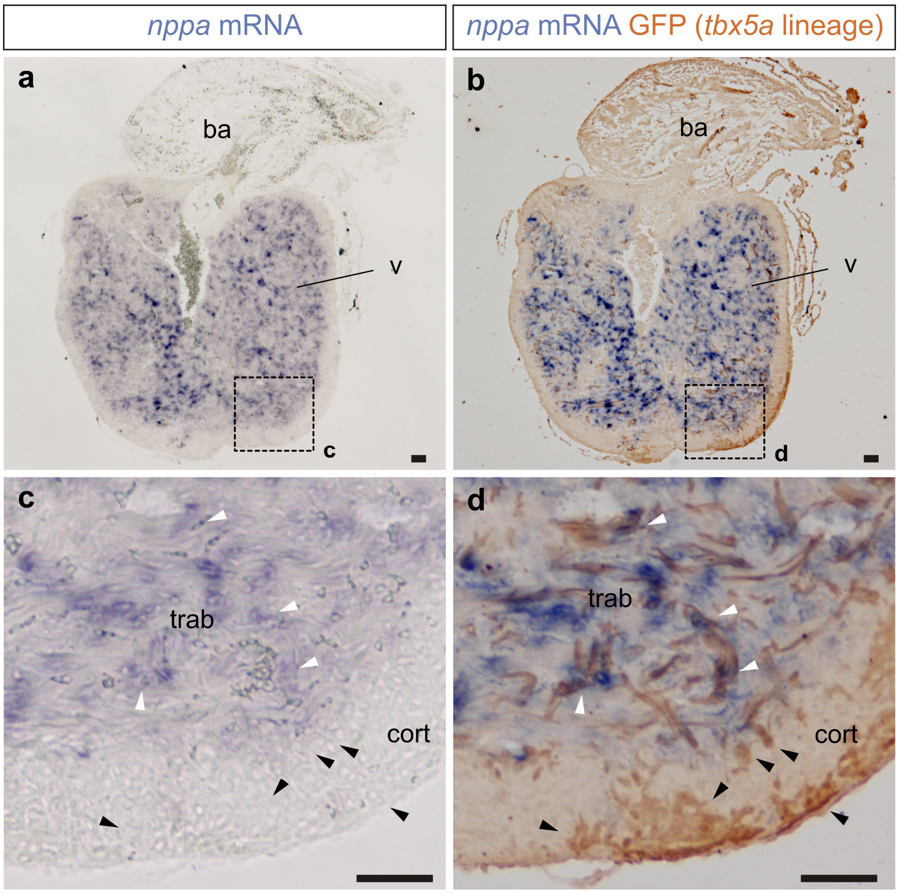

Supplementary Figure 17. *tbx5a*-derived cardiomyocytes within the cortical layer are *nppa* negative. *nppa* mRNA *in situ* hybridisation (**a**) on sagittal sections of *tbx5a*:*mCherryp-2a-CreER*^{T2};*ubb*:*loxP-lacZ-loxP-GFP* double transgenic animals, recombined before injury and fixed at 90 days postinjury (dpi) followed by GFP immunofluorescence (**b**) *nppa* is expressed in the trabecular layer (white arrowheads). In the cortical region, *tbx5a*-derived GFP$^+$ cells are visible, which are not positive for the trabecular marker *nppa* (black arrowheads, n=7/7). **c** and **d** are a zoomed with of boxed areas in **a** and **b.** ba, bulbus arteriosus; cort, cortical layer; trab, trabecular layer; v, ventricle. Scale bars, 50 µm

Number	Name	Sequence
1	pTarBAC_HA1_iTol2_F	gcgtaagcggggcacatttcattacctctttctccgcacccgacatagatCCCTGCTCGAGCCGGGCCCAAGTG
2	pTarBAC_HA2_iTol2_R	gcggggcatgactattggcgcgccggatcgatccttaattaagtctactaATTATGATCCTCTAGATCAGATC
3	tbx5_HA1_mCherry_F	ctttttgtttctgtatttaggcctcacggtagacatcgtacaggcctctccACCATGGTGAGCAAGGGC
4	tbx5a_HA2_kanFRT_R	ttcgctgtcactgggagagttttggagccgaaaggtgtcttcactgtccgcGGAGGCTACCATGGAGAAG
5	pTarBAC_HA1_Cryst_F	gcgtaagcggggcacatttcattacctctttctccgcacccgacatagatTACCGGGCCCCCCCTCGAGTCC
6	tbx5_HA1_CreERT2_F	ctttttgtttctgtatttaggcctcacggtagacatcgtacaggcctctccaccatgTCCAACCTGCTGACTGTGCACC
7	vmhcl_HA1_loxP_F	atgtcctgtactgcttctaacaagttcttcttttccataatttaaggttgACCGGTGGATCCACTATAAC
8	vmhcl_HA2_kanFRT_R	ttccgcaggtaaggcgctgcggccccaaaaacagacatttcagcatcgccGGAGGCTACCATGGAGAAG

Supplementary Table 1. Sequence of the primers used for BAC Recombineering. Red, homology arms. Green, minimal kozak sequence. Capital letters, overlapping with template sequence
