# Supplementary material for: Tbx5a lineage tracing shows cardiomyocyte plasticity during zebrafish heart regeneration
Source: Nat Commun. 2018 Jan 30;9:428. doi: 10.1038/s41467-017-02650-6 (PMC5789846; doi:10.1038/s41467-017-02650-6)
Supplement: Supplementary file 5 — Supplementary Data 2 [file 41467_2017_2650_MOESM5_ESM.pdf]

Supplementary Data 2. Sequence of the plasmid used for recombineering of the tbx5a:tdTomato BAC.

```
LOCUS       tdTomato_tbx5a_homology_arms       7528 bp ds-DNA     linear
09-AUG-2017



COMMENT     ApEinfo:methylated:1
FEATURES             Location/Qualifiers
     misc_feature    675..1252
                     /label=5'arm
                     /ApEinfo_fwdcolor=#ff0000
                     /ApEinfo_revcolor=green
                     /ApEinfo_graphicformat=arrow_data {{0 1 2 0 0 -1} {} 0}
                     width 5 offset 0
     misc_feature    4847..5313
                     /label=5'arm(1)
                     /ApEinfo_label=5'arm
                     /ApEinfo_fwdcolor=#ff0000
                     /ApEinfo_revcolor=green
                     /ApEinfo_graphicformat=arrow_data {{0 1 2 0 0 -1} {} 0}
                     width 5 offset 0
     misc_feature    3612..4412
                     /label=NeoR/KanR
                     /ApEinfo_fwdcolor=#00ff00
                     /ApEinfo_revcolor=green
                     /ApEinfo_graphicformat=arrow_data {{0 1 2 0 0 -1} {} 0}
                     width 5 offset 0
     misc_feature    complement(6540..7400)
                     /label=AmpR
                     /ApEinfo_fwdcolor=#ff00ff
                     /ApEinfo_revcolor=green
                     /ApEinfo_graphicformat=arrow_data {{0 1 2 0 0 -1} {} 0}
                     width 5 offset 0
     misc_feature    4732..4766
                     /label=FRT
                     /ApEinfo_fwdcolor=#ff8000
                     /ApEinfo_revcolor=green
                     /ApEinfo_graphicformat=arrow_data {{0 1 2 0 0 -1} {} 0}
                     width 5 offset 0
     misc_feature    2938..2972
                     /label=FRT(1)
                     /ApEinfo_label=FRT
                     /ApEinfo_fwdcolor=#ff8000
                     /ApEinfo_revcolor=green
                     /ApEinfo_graphicformat=arrow_data {{0 1 2 0 0 -1} {} 0}
                     width 5 offset 0
     misc_feature    1255..2685
                     /label=tdTomato
                     /ApEinfo_fwdcolor=#ff8000
                     /ApEinfo_revcolor=#ff8000
                     /ApEinfo_graphicformat=arrow_data {{0 1 2 0 0 -1} {} 0}
                     width 5 offset 0
ORIGIN
        1 CTAAATTGTA AGCGTTAATA TTTTGTTAAA ATTCGCGTTA AATTTTTGTT AAATCAGCTC
       61 ATTTTTTAAC CAATAGGCCG AAATCGGCAA AATCCCTTAT AAATCAAAAG AATAGACCGA
      121 GATAGGGTTG AGTGTTGTTC CAGTTTGGAA CAAGAGTCCA CTATTAAAGA ACGTGGACTC
      181 CAACGTCAAA GGGCGAAAAA CCGTCTATCA GGGCGATGGC CCACTACGTG AACCATCACC
      241 CTAATCAAGT TTTTTGGGGT CGAGGTGCCG TAAAGCACTA AATCGGAACC CTAAAGGGAG
      301 CCCCCGATTT AGAGCTTGAC GGGGAAAGCC GGCGAACGTG GCGAGAAAGG AAGGGAAGAA
      361 AGCGAAAGGA GCGGGCGCTA GGGCGCTGGC AAGTGTAGCG GTCACGCTGC GCGTAACCAC
      421 CACACCCGCC GCGCTTAATG CGCCGCTACA GGGCGCGTCC CATTCGCCAT TCAGGCTGCG
      481 CAACTGTTGG GAAGGGCGAT CGGTGCGGGC CTCTTCGCTA TTACGCCAGC TGGCGAAAGG
      541 GGGATGTGCT GCAAGGCGAT TAAGTTGGGT AACGCCAGGG TTTTCCCAGT CACGACGTTG
      601 TAAAACGACG GCCAGTGAAT TGTAATACGA CTCACTATAG GGCGAATTGG GTACCGGGCC
      661 CCCCCTCGAG GTCGACTTAT AGGCCAGCAA TCGCTTCAGA TTCATGAACT ATCGGTGTAC
      721 AAACTATTAG TGATACACAC ATAATCATCA ACTGTTGGTT ATTTAATAGA AAACCAGCTA
```

```
781 TCGTTCACCA GTGCATTTGT TTTCCTGAAC TACAATAAAG TTTTAATGGA GGCCCGTGTT
841 TTGATTTTAT CCACAGACTG GTTTTAAGAA AATATAAGCC GCATTGAAAC CTCTTTGTGA
901 TGTGCATTTT AATGTGCGTG TTTGGTGCAA AGTTGAGTCC ACCTGAATAA TGTTTGTGCA
961 GAGAGCTCCA AATTATAAAA TGTTTAATAG GATCAGAGTT TTATTCTTAT TGATAAATGT
1021 TTGCTTTTTT CTAATTAACA CTGCTGTACT GTAATAAAGT TAATTTCGGT TGTTGTTGAA
1081 TGTTTTCATT AAATCAGATT CAAGAGCACA CTGATAAGTT ATATCACCGT AATCAAATGC
1141 ATACGGGCGT CTTGAAGTGA TGCAATCTGA ACTAAACTGC AGAAATATTT GATGTAAAAT
1201 CTTTTTGTTT CTGTATTTAG GCCTCACGGT AGACATCGTA CAGGCCTCTC CGCCatggtg
1261 agcaagggcg aggaggtcat caaagagttc atgcgcttca aggtgcgcat ggagggctcc
1321 atgaacggcc acgagttcga gatcgagggc gagggcgagg gccgccccta cgagggcacc
1381 cagaccgcca agctgaaggt gaccaagggc ggccccctgc ccttcgcctg ggacatcctg
1441 tccccccagt tcatgtacgg ctccaaggcg tacgtgaagc accccgccga catccccgat
1501 tacaagaagc tgtccttccc cgagggcttc aagtgggagc gcgtgatgaa cttcgaggac
1561 ggcggtctgg tgaccgtgac ccaggactcc tccctgcagg acggcacgct gatctacaag
1621 gtgaagatgc gcggcaccaa cttccccccc gacggccccg taatgcagaa gaagaccatg
1681 ggctgggagg cctccaccga gcgcctgtac cccgcgacg gcgtgctgaa gggcgagatc
1741 caccaggccc tgaagctgaa ggacggcggc cactacctgg tggagttcaa gaccatctac
1801 atggccaaga agcccgtgca actgcccggc tactactacg tggacaccaa gctggacatc
1861 acctcccaca acgaggacta caccatcgtg gaacagtacg agcgctccga gggccgccac
1921 cacctgttcc tggggcatgg caccggcagc accggcagcg gcagctccgg caccgcctcc
1981 tccgaggaca acaacatggc cgtcatcaaa gagttcatgc gcttcaaggt gcgcatggag
2041 ggctccatga acggccacga gttcgagatc gagggcgagg gcgagggccg cccctacgag
2101 ggcacccaga ccgccaagct gaaggtgacc aagggcggcc ccctgccctt cgcctgggac
2161 atcctgtccc cccagttcat gtacggctcc aaggcgtacg tgaagcaccc cgccgacatc
2221 cccgattaca agaagctgtc cttccccgag ggcttcaagt gggagcgcgt gatgaacttc
2281 gaggacggcg gtctggtgac cgtgacccag gactcctccc tgcaggacgg cacgctgatc
2341 tacaaggtga agatgcgcgg caccaacttc ccccccgacg gccccgtaat gcagaagaag
2401 accatgggct gggaggcctc caccgagcgc ctgtaccccc gcgacggcgt gctgaagggc
2461 gagatccacc aggccctgaa gctgaaggac ggcggccgct acctggtgga gttcaagacc
2521 atctacatgg ccaagaagcc cgtgcaactg cccggctact actacgtgga caccaagctg
2581 gacatcacct cccacaacga ggactacacc atcgtggaac agtacgagcg ctccgagggc
2641 cgccaccacc tgttcctgta cggcatggac gagctgtaca agtaaAGCGG CCGCGACTCT
2701 AGATCATAAT CAGCCATACC ACATTTGTAG AGGTTTTACT TGCTTTAAAA AACCTCCCAC
2761 ACCTCCCCCT GAACCTGAAA CATAAAATGA ATGCAATTGT TGTTGTTAAC TTGTTTATTG
2821 CAGCTTATAA TGGTTACAAA TAAAGCAATA GCATCACAAA TTTCACAAAT AAAGCATTTT
2881 TTTCACTGCA TTCTAGTTGT GGTTTGTCCA AACTCATCAA GCTTGATATC GAATTCCGAA
2941 GTTCCTATTC TCTAGAAAGT ATAGGAACTT CAGGTCTGAA GAGGAGTTTA CGTCCAGCCA
3001 AGCTAGCTTG GCTGCAGGTC GTCGAAATTC TACCGGGTAG GGGAGGCGCT TTTCCCAAGG
3061 CAGTCTGGAG CATGCGCTTT AGCAGCCCCG CTGGGCACTT GGCGCTACAC AAGTGGCCTC
3121 TGGCCTCGCA CACATTCCAC ATCCACCGGT AGGCGCCAAC CGGCTCCGTT CTTTGGTGGC
3181 CCCTTCGCGC CACCTTCTAC TCCTCCCCTA GTCAGGAAGT TCCCCCCCGC CCCGCAGCTC
3241 GCGTCGTGCA GGACGTGACA AATGGAAGTA GCACGTCTCA CTAGTCTCGT GCAGATGGAC
3301 AGCACCGCTG AGCAATGGAA GCGGGTAGGC CTTTGGGGCA GCGGCCAATA GCAGCTTTGC
3361 TCCTTCGCTT TCTGGGCTCA GAGGCTGGGA AGGGGTGGGT CCGGGGGCGG GCTCAGGGGC
3421 GGGCTCAGGG GCGGGGCGGG CGCCCGAAGG TCCTCCGGAG GCCCGGCATT CTGCACGCTT
3481 CAAAAGCGCA CGTCTGCCGC GCTGTTCTCC TCTTCCTCAT CTCCGGGCCT TTCGACCTGC
3541 AGCCTGTTGA CAATTAATCA TCGGCATAGT ATATCGGCAT AGTATAATAC GACAAGGTGA
3601 GGAACTAAAC CATGGGATCG GCCATTGAAC AAGATGGATT GCACGCAGGT TCTCCGGCCG
3661 CTTGGGTGGA GAGGCTATTC GGCTATGACT GGGCACAACA GACAATCGGC TGCTCTGATG
3721 CCGCCGTGTT CCGGCTGTCA GCGCAGGGGC GCCCGGTTCT TTTTGTCAAG ACCGACCTGT
3781 CCGGTGCCCT GAATGAACTG CAGGACGAGG CAGCGCGGCT ATCGTGGCTG GCCACGACGG
3841 GCGTTCCTTG CGCAGCTGTG CTCGACGTTG TCACTGAAGC GGGAAGGGAC TGGCTGCTAT
3901 TGGGCGAAGT GCCGGGGCAG GATCTCCTGT CATCTCACCT TGCTCCTGCC GAGAAAGTAT
3961 CCATCATGGC TGATGCAATG CGGCGGCTGC ATACGCTTGA TCCGGCTACC TGCCCATTCG
4021 ACCACCAAGC GAAACATCGC ATCGAGCGAG CACGTACTCG GATGGAAGCC GGTCTTGTCG
4081 ATCAGGATGA TCTGGACGAA GAGCATCAGG GGCTCGCGCC AGCCGAACTG TTCGCCAGGC
4141 TCAAGGCGCG CATGCCCGAC GGCGATGATC TCGTCGTGAC CCATGGCGAT GCCTGCTTGC
4201 CGAATATCAT GGTGGAAAAT GGCCGCTTTT CTGGATTCAT CGACTGTGGC CGGCTGGGTG
4261 TGGCGGACCG CTATCAGGAC ATAGCGTTGG CTACCCGTGA TATTGCTGAA GAGCTTGGCG
4321 GCGAATGGGC TGACCGCTTC CTCGTGCTTT ACGGTATCGC CGCTCCCGAT TCGCAGCGCA
4381 TCGCCTTCTA TCGCCTTCTT GACGAGTTCT TCTGAGGGGA TCAATTCTCT AGAGCTCGCT
4441 GATCAGCCTC GACTGTGCCT TCTAGTTGCC AGCCATCTGT TGTTTGCCCC TCCCCCGTGC
4501 CTTCCTTGAC CCTGGAAGGT GCCACTCCCA CTGTCCTTTC CTAATAAAAT GAGGAAATTG
4561 CATCGCATTG TCTGAGTAGG TGTCATTCTA TTCTGGGGGG TGGGGTGGGG CAGGACAGCA
4621 AGGGGGAGGA TTGGGAAGAC AATAGCAGGC ATGCTGGGGA TGCGGTGGGC TCTATGGCTT
4681 CTGAGGCGGA AAGAACCAGC TGGGGCTCGA CTAGAGCTTG CGGAACCCTT CGAAGTTCCT
4741 ATTCTCTAGA AAGTATAGGA ACTTCATCAG TCAGGTACAT AATATAACTT CGTATAATGT
4801 ATGCTATACG AAGTTATTAG GTGGATCCAC TAGTTCTAGA GCGGCCGCGG ACAGTGAAGA
```

4861 CACCTTTCGG CTCCAAAACT CTCCCAGTGA CAGCGAACCA AAAGATTTAC AAAACGAAGG
4921 AAAATCAGAC AAACAGAATG CAGCCGTCAG CAAATCCCCG TCATCGCAGA CTACATACAT
4981 TCAACAGGTA ACGTCAACTA GTGCCTCGAT GGTGCGTGTG CGTGTGTGTG TGTGTGTGCA
5041 AGTGCTTTAT GGTAAACTAA ACCTGATAAA CACCTCTCTT TTAGAATTAT TTCGATCATT
5101 TTCATATTCA AAAACTTTTC ATTTAGCTTT AGGTTAGTTA TTTATATTAA AAACACTAGT
5161 ATATAAGTAA CTAAAATAAA CATTCAACAG ATAACTTTAA CTAGTGTCTT GGCGTGTGCG
5221 TGTGTGTGTG CCCAGAGTCT TTAGTAATTT TAAGTATTAA TAAAAATTTA AGAAATGCAA
5281 TATGTTTTCA CGTGGGCTAC AGTTATTTAT ATAccgcggT GGAGCTCCAG CTTTTGTTCC
5341 CTTTAGTGAG GGTTAATTTC GAGCTTGGCG TAATCATGGT CATAGCTGTT TCCTGTGTGA
5401 AATTGTTATC CGCTCACAAT TCCACACAAC ATACGAGCCG GAAGCATAAA GTGTAAAGCC
5461 TGGGGTGCCT AATGAGTGAG CTAACTCACA TTAATTGCGT TGCGCTCACT GCCCGCTTTC
5521 CAGTCGGGAA ACCTGTCGTG CCAGCTGCAT TAATGAATCG GCCAACGCGC GGGGAGAGGC
5581 GGTTTGCGTA TTGGGCGCTC TTCCGCTTCC TCGCTCACTG ACTCGCTGCG CTCGGTCGTT
5641 CGGCTGCGGC GAGCGGTATC AGCTCACTCA AAGGCGGTAA TACGGTTATC CACAGAATCA
5701 GGGGATAACG CAGGAAAGAA CATGTGAGCA AAAGGCCAGC AAAAGGCCAG GAACCGTAAA
5761 AAGGCCGCGT TGCTGGCGTT TTTCCATAGG CTCCGCCCCC CTGACGAGCA TCACAAAAAT
5821 CGACGCTCAA GTCAGAGGTG GCGAAACCCG ACAGGACTAT AAAGATACCA GGCGTTTCCC
5881 CCTGGAAGCT CCCTCGTGCG CTCTCCTGTT CCGACCCTGC CGCTTACCGG ATACCTGTCC
5941 GCCTTTCTCC CTTCGGGAAG CGTGGCGCTT TCTCATAGCT CACGCTGTAG GTATCTCAGT
6001 TCGGTGTAGG TCGTTCGCTC CAAGCTGGGC TGTGTGCACG AACCCCCCGT TCAGCCCGAC
6061 CGCTGCGCCT TATCCGGTAA CTATCGTCTT GAGTCCAACC CGGTAAGACA CGACTTATCG
6121 CCACTGGCAG CAGCCACTGG TAACAGGATT AGCAGAGCGA GGTATGTAGG CGGTGCTACA
6181 GAGTTCTTGA AGTGGTGGCC TAACTACGGC TACACTAGAA GAACAGTATT TGGTATCTGC
6241 GCTCTGCTGA AGCCAGTTAC CTTCGGAAAA AGAGTTGGTA GCTCTTGATC CGGCAAACAA
6301 ACCACCGCTG GTAGCGGTGG TTTTTTTGTT TGCAAGCAGC AGATTACGCG CAGAAAAAAA
6361 GGATCTCAAG AAGATCCTTT GATCTTTTCT ACGGGGTCTG ACGCTCAGTG GAACGAAAAC
6421 TCACGTTAAG GGATTTTGGT CATGAGATTA TCAAAAAGGA TCTTCACCTA GATCCTTTTA
6481 AATTAAAAAT GAAGTTTTAA ATCAATCTAA AGTATATATG AGTAAACTTG GTCTGACAGT
6541 TACCAATGCT TAATCAGTGA GGCACCTATC TCAGCGATCT GTCTATTTCG TTCATCCATA
6601 GTTGCCTGAC TCCCCGTCGT GTAGATAACT ACGATACGGG AGGGCTTACC ATCTGGCCCC
6661 AGTGCTGCAA TGATACCGCG AGACCCACGC TCACCGGCTC CAGATTTATC AGCAATAAAC
6721 CAGCCAGCCG GAAGGGCCGA GCGCAGAAGT GGTCCTGCAA CTTTATCCGC CTCCATCCAG
6781 TCTATTAATT GTTGCCGGGA AGCTAGAGTA AGTAGTTCGC CAGTTAATAG TTTGCGCAAC
6841 GTTGTTGCCA TTGCTACAGG CATCGTGGTG TCACGCTCGT CGTTTGGTAT GGCTTCATTC
6901 AGCTCCGGTT CCCAACGATC AAGGCGAGTT ACATGATCCC CCATGTTGTG CAAAAAAGCG
6961 GTTAGCTCCT TCGGTCCTCC GATCGTTGTC AGAAGTAAGT TGGCCGCAGT GTTATCACTC
7021 ATGGTTATGG CAGCACTGCA TAATTCTCTT ACTGTCATGC CATCCGTAAG ATGCTTTTCT
7081 GTGACTGGTG AGTACTCAAC CAAGTCATTC TGAGAATAGT GTATGCGGCG ACCGAGTTGC
7141 TCTTGCCCGG CGTCAATACG GGATAATACC GCGCCACATA GCAGAACTTT AAAAGTGCTC
7201 ATCATTGGAA AACGTTCTTC GGGGCGAAAA CTCTCAAGGA TCTTACCGCT GTTGAGATCC
7261 AGTTCGATGT AACCCACTCG TGCACCCAAC TGATCTTCAG CATCTTTTAC TTTCACCAGC
7321 GTTTCTGGGT GAGCAAAAAC AGGAAGGCAA AATGCCGCAA AAAAGGGAAT AAGGGCGACA
7381 CGGAAATGTT GAATACTCAT ACTCTTCCTT TTTCAATATT ATTGAAGCAT TTATCAGGGT
7441 TATTGTCTCA TGAGCGGATA CATATTTGAA TGTATTTAGA AAAATAAACA AATAGGGGTT
7501 CCGCGCACAT TTCCCCGAAA AGTGCCAC
//
