# Supplementary material for: Tbx5a lineage tracing shows cardiomyocyte plasticity during zebrafish heart regeneration
Source: Nat Commun. 2018 Jan 30;9:428. doi: 10.1038/s41467-017-02650-6 (PMC5789846; doi:10.1038/s41467-017-02650-6)
Supplement: Supplementary file 6 — Supplementary Data 3 [file 41467_2017_2650_MOESM6_ESM.pdf]

Supplementary Data 3. Sequence of the plasmid containing
mCherry-p2a-CreERT2-flp-kan-flp used as a template for recombineering.

```
LOCUS       Exported                7593 bp ds-DNA     circular SYN 30-AGO-2016
DEFINITION  synthetic circular DNA


REFERENCE   1  (bases 1 to 7593)
  AUTHORS   .
  TITLE     Direct Submission
  JOURNAL   Exported martes, 30 de ago de 2016 from SnapGene Viewer 3.0.3
            http://www.snapgene.com
FEATURES             Location/Qualifiers
     source          1..7593
                     /organism="synthetic DNA construct"
                     /mol_type="other DNA"
     misc_feature    413..435
                     /label=SP6
                     /note="SP6"
     misc_feature    439..1146
                     /label=mCherry
                     /note="mCherry"
     misc_feature    1147..1212
                     /label=P2A
                     /note="P2A"
     misc_feature    1213..3198
                     /label=CreERT2
                     /note="CreERT2"
     misc_feature    3226..3464
                     /label=SV40 poliA
                     /note="SV40 poliA"
     misc_feature    3509..3543
                     /label=FRT
                     /note="FRT"
     misc_feature    3798..3936
                     /label=SV40 poliA(1)
                     /note="SV40 poliA(1)"
     misc_feature    3987..4779
                     /label=KanR
                     /note="KanR"
     misc_feature    4860..4894
                     /label=FRT(1)
                     /note="FRT(1)"
ORIGIN
        1 tcgcgcgttt cggtgatgac ggtgaaaacc tctgacacat gcagctcccg gagacggtca
       61 cagcttgtct gtaagcggat gccgggagca gacaagcccg tcagggcgcg tcagcgggtg
      121 ttggcgggtg tcggggctgg cttaactatg cggcatcaga gcagattgta ctgagagtgc
      181 accatatgcg gtgtgaaata ccgcacagat gcgtaaggag aaaataccgc atcaggcgcc
      241 attcgccatt caggctgcgc aactgttggg aagggcgatc ggtgcgggcc tcttcgctat
      301 tacgccagct ggcgaaaggg ggatgtgctg caaggcgatt aagttgggta acgccagggt
      361 tttcccagtc acgacgttgt aaaacgacgg ccagtgccaa gctcggcgcg ccatttaggt
      421 gacactatag aagagaccat ggtgagcaag ggcgaggagg ataacatggc catcatcaag
      481 gagttcatgc gcttcaaggt gcacatggag ggctccgtga acggccacga gttcgagatc
      541 gagggcgagg gcgagggccg cccctacgag ggcacccaga ccgccaagct gaaggtgacc
      601 aagggtggcc ccctgccctt cgcctgggac atcctgtccc ctcagttcat gtacggctcc
      661 aaggcctacg tgaagcaccc cgccgacatc cccgactact tgaagctgtc cttccccgag
      721 ggcttcaagt gggagcgcgt gatgaacttc gaggacggcg gcgtggtgac cgtgacccag
      781 gactcctccc tgcaggacgg cgagttcatc tacaaggtga agctgcgcgg caccaacttc
      841 ccctccgacg gccccgtaat gcagaagaag accatgggct gggaggcctc ctccgagcgg
      901 atgtaccccg aggacggcgc cctgaagggc gagatcaagc agaggctgaa gctgaaggac
      961 ggcggccact acgacgctga ggtcaagacc acctacaagg ccaagaagcc cgtgcagctg
     1021 cccggcgcct acaacgtcaa catcaagttg gacatcacct cccacaacga ggactacacc
     1081 atcgtggaac agtacgaacg cgccgagggc cgccactcca ccggcggcat ggacgagctg
     1141 tacaagggaa gcggagctac taacttcagc ctgctgaagc aggctggaga cgtggaggag
     1201 aaccctggac ctgctagctc caacctgctg actgtgcacc aaaacctgcc tgccctccct
     1261 gtggatgcca cctctgatga agtcaggaag aacctgatgg acatgttcag ggacaggcag
     1321 gccttctctg aacacacctg gaagatgctc ctgtctgtgt gcagatcctg ggctgcctgg
     1381 tgcaagctga acaacaggaa atggttccct gctgaacctg aggatgtgag ggactacctc
```

```
1441 ctgtacctgc aagccagagg cctggctgtg aagaccatcc aacagcacct gggccagctc
1501 aacatgctgc acaggagatc tggcctgcct cgcccttctg actccaatgc tgtgtccctg
1561 gtgatgagga gaatcagaaa ggagaatgtg gatgctgggg agagagccaa gcaggccctg
1621 gcctttgaac gcactgactt tgaccaagtc agatccctga tggagaactc tgacagatgc
1681 caggacatca ggaacctggc cttcctgggc attgcctaca acaccctgct gcgcattgcc
1741 gaaattgcca gaatcagagt gaaggacatc tcccgcaccg atggtgggag aatgctgatc
1801 cacattggca ggaccaagac cctggtgtcc acagctggtg tggagaaggc cctgtccctg
1861 ggggttacca agctggtgga gagatggatc tctgtgtctg gtgtggctga tgaccccaac
1921 aactacctgt tctgccgggt cagaaagaat ggtgtggctg ccccttctgc cacctcccaa
1981 ctgtccaccc gggccctgga agggatcttt gaggccaccc accgcctgat ctatggtgcc
2041 aaggatgact ctgggcagag atacctggcc tggtctggcc actctgccag agtgggtgct
2101 gccagggaca tggccagggc tggtgtgtcc atccctgaaa tcatgcaggc tggtggctgg
2161 accaatgtga acattgtgat gaactacatc agaaacctgg actctgagac tggggctatg
2221 gtgaggctgc ttgaggatgg ggacctcgag ccatctgctg gagacatgag agctgccaac
2281 ctttggccaa gcccgctcat gatcaaacgc tctaagaaga acagcctggc cttgtccctg
2341 acggccgacc agatggtcag tgccttgttg gatgctgagc cccccatact ctattccgag
2401 tatgatccta ccagaccctt cagtgaagct tcgatgatgg gcttactgac caacctggca
2461 gacagggagc tggttcacat gatcaactgg gcgaagaggg tgccaggctt tgtggatttg
2521 accctccatg atcaggtcca ccttctagaa tgtgcctggc tagagatcct gatgattggt
2581 ctcgtctggc gctccatgga gcacccagtg aagctactgt ttgctcctaa cttgctcttg
2641 gacaggaacc agggaaaatg tgtagagggc atggtggaga tcttcgacat gctgctggct
2701 acatcatctc ggttccgcat gatgaatctg cagggagagg agtttgtgtg cctcaaatct
2761 attattttgc ttaattctgg agtgtacaca tttctgtcca gcaccctgaa gtctctggaa
2821 gagaaggacc atatccaccg agtcctggac aagatcacag acactttgat ccacctgatg
2881 gccaaggcag gcctgaccct gcagcagcag caccagcggc tggcccagct cctcctcatc
2941 ctctcccaca tcaggcacat gagtaacaaa ggcatggagc atctgtacag catgaagtgc
3001 aagaacgtgg tgcccctcta tgacctgctg ctggaggcgg cggacgccca ccgcctacat
3061 gcgcccacta gccgtggagg ggcatccgtg gaggagacgg accaaagcca cttggccact
3121 gcgggctcta cttcatcgca ttccttgcaa aagtattaca tcacgggggga ggcagagggt
3181 ttccctgcca cagcttgatg aagatctgag ctcgaattcg cccttaggcc tgcaggatca
3241 taatcagcca taccacattt gtagaggttt tacttgcttt aaaaaacctc ccacacctcc
3301 ccctgaacct gaaacataaa atgaatgcaa ttgttgttgt taacttgttt attgcagctt
3361 ataatggtta caaataaagc aatagcatca caaatttcac aaataaagca tttttttcac
3421 tgcattctag ttgtggtttg tccaaactca tcaatgtatc ttaaactagg ggggatctgc
3481 tagcccggat ctagataact gatcagcttg aagttcctat actttctaga gaataggaac
3541 ttcggaatag gaacttcaag atccccctgg cgaaaggggg atgtgctgca aggcgattaa
3601 gttgggtaac gccagggttt tcgtcaggtg gcacttttcg gggaaatgtg cgcggaaccc
3661 ctatttgttt atttttctaa atacattcaa atatgtatcc gctcatgaga caataaccct
3721 gataaatgct tcaataatat tgaaaaagga agagtcctga ggcggaaaga accagctgtg
3781 gaatgtgtgt cagttagggt gtggaaagtc cccaggctcc ccagcaggca gaagtatgca
3841 aagcatgcat ctcaattagt cagcaaccag gtgtggaaag tccccaggct ccccagcagg
3901 cagaagtatg caaagcatgc atctcaatta gtcagtaacc atagtcccgca aagatcgatc
3961 aagagacagg atgaggatcg tttcgcatga ttgaacaaga tggattgcac gcaggttctc
4021 cggccgcttg ggtggagagg ctattcggct atgactgggc acaacagaca atcggctgct
4081 ctgatgccgc cgtgttccgg ctgtcagcgc aggggcgccc ggttcttttt gtcaagaccg
4141 acctgtccgg tgccctgaat gaactgcaag acgaggcagc gcggctatcg tggctggcca
4201 cgacgggcgt tccttgcgca gctgtgctcg acgttgtcac tgaagcggga agggactggc
4261 tgctattggg cgaagtgccg gggcaggatc tcctgtcatc tcaccttgct cctgccgaga
4321 aagtatccat catggctgat gcaatgcggc ggctgcatac gcttgatccg gctacctgcc
4381 cattcgacca ccaagcgaaa catcgcatcg agcgagcacg tactcggatg gaagccggtc
4441 ttgtcgatca ggatgatctg gacgaagagc atcaggggct cgcgccagcc gaactgttcg
4501 ccaggctcaa ggcgagcatg cccgacggcg aggatctcgt cgtgacccat ggcgatgcct
4561 gcttgccgaa tatcatggtg gaaaatggcc gcttttctgg attcatcgac tgtggccggc
4621 tgggtgtggc ggaccgctat caggacatag cgttggctac ccgtgatatt gctgaagagc
4681 ttggcggcga atgggctgac cgcttcctcg tgctttacgg tatcgccgct cccgattcgc
4741 agcgcatcgc cttctatcgc cttcttgacg agttcttctg aggggatcgg caataaaaag
4801 acagaataaa acgcacgggt gttgggtcgt ttgttcggat ccgagcttca aaagcgctct
4861 gaagttccta tactttctag agaataggaa cttcggaata gtaacttctc catggtagcc
4921 tccaaaaaag cctcctcact acttctggac tagtgcggcc gccagagctg gccactgctt
4981 tgggagcctt cctgggcctc ctggcagtgg cagcaatggc aggccctaac tttccccaga
5041 tagacacccc caacatgcta cctgcccacc atcgccaaaa gagagactgg atttggaatc
5101 aaatgcacat cgatgaagag aaaaatgaat cgctgcccca ctatgtggga aaggtaagcc
5161 tcgcgctcca gggtgatggc cattatcaac actggaccat agtcatggcc atgtagccaa
5221 gatggtggtg aataagattg gttatcattg gtggtgcggg gggacggaga ggaggcaaag
5281 aacagggtgg taataatggt gaaagtgaca tgtgtgctga tggttgtgat aggaaagaga
5341 aattaattaa gagctcgaat tcgtaatcat ggtcatagct gtttcctgtg tgaaattgtt
5401 atccgctcac aattccacac aacatacgag ccggaagcat aaagtgtaaa gcctggggtg
5461 cctaatgagt gagctaactc acattaattg cgttgcgctc actgcccgct ttccagtcgg
```

```
5521 gaaacctgtc gtgccagctg cattaatgaa tcggccaacg cgcggggaga ggcggtttgc
5581 gtattgggcg ctcttccgct tcctcgctca ctgactcgct gcgctcggtc gttcggctgc
5641 ggcgagcggt atcagctcac tcaaaggcgg taatacggtt atccacagaa tcaggggata
5701 acgcaggaaa gaacatgtga gcaaaaggcc agcaaaaggc caggaaccgt aaaaaggccg
5761 cgttgctggc gtttttccat aggctccgcc cccctgacga gcatcacaaa aatcgacgct
5821 caagtcagag gtggcgaaac ccgacaggac tataaagata ccaggcgttt ccccctggaa
5881 gctccctcgt gcgctctcct gttccgaccc tgccgcttac cggatacctg tccgcctttc
5941 tcccttcggg aagcgtggcg ctttctcaaa gctcacgctg taggtatctc agttcggtgt
6001 aggtcgttcg ctccaagctg ggctgtgtgc acgaaccccc cgttcagccc gaccgctgcg
6061 ccttatccgg taactatcgt cttgagtcca acccggtaag acacgactta tcgccactgg
6121 cagcagccac tggtaacagg attagcagag cgaggtatgt aggcggtgct acagagttct
6181 tgaagtggtg gcctaactac ggctacacta gaagaacagt atttggtatc tgcgctctgc
6241 tgaagccagt taccttcgga aaaagagttg gtagctcttg atccggcaaa caaaccaccg
6301 ctggtagcgg tggttttttt gtttgcaagc agcagattac gcgcagaaaa aaaggatctc
6361 aagaagatcc tttgatcttt tctacggggt ctgacgctca gtggaacgaa aactcacgtt
6421 aagggatttt ggtcatgaga ttatcaaaaa ggatcttcac ctagatcctt ttaaattaaa
6481 aatgaagttt taaatcaatc taaagtatat atgagtaaac ttggtctgac agttaccaat
6541 gcttaatcag tgaggcacct atctcagcga tctgtctatt tcgttcatcc atagttgcct
6601 gactccccgt cgtgtagata actacgatac gggagggctt accatctggc cccagtgctg
6661 caatgatacc gcgagaccca cgctcaccgg ctccagattt atcagcaata aaccagccag
6721 ccggaagggc cgagcgcaga agtggtcctg caactttatc cgcctccatc cagtctatta
6781 attgttgccg ggaagctaga gtaagtagtt cgccagttaa tagtttgcgc aacgttgttg
6841 ccattgctac aggcatcgtg gtgtcacgct cgtcgtttgg tatggcttca ttcagctccg
6901 gttcccaacg atcaaggcga gttacatgat cccccatgtt gtgcaaaaaa gcggttagct
6961 ccttcggtcc tccgatcgtt gtcagaagta agttggccgc agtgttatca ctcatggtta
7021 tggcagcact gcataattct cttactgtca tgccatccgt aagatgcttt tctgtgactg
7081 gtgagtactc aaccaagtca ttctgagaat agtgtatgcg gcgaccgagt tgctcttgcc
7141 cggcgtcaat acgggataat accgcgccac atagcagaac tttaaaagtg ctcatcattg
7201 gaaaacgttc ttcggggcga aaactctcaa ggatcttacc gctgttgaga tccagttcga
7261 tgtaacccac tcgtgcaccc aactgatctt cagcatcttt tactttcacc agcgtttctg
7321 ggtgagcaaa aacaggaagg caaaatgccg caaaaaaggg aataagggcg acacggaaat
7381 gttgaatact catactcttc ctttttcaat attattgaag catttatcag ggttattgtc
7441 tcatgagcgg atacatattt gaatgtattt agaaaaataa acaaataggg gttccgcgca
7501 catttccccg aaaagtgcca cctgacgtct aagaaaccat tattatcatg acattaacct
7561 ataaaaatag gcgtatcacg aggccctttc gtc
//
```
