# Supplementary material for: Tbx5a lineage tracing shows cardiomyocyte plasticity during zebrafish heart regeneration
Source: Nat Commun. 2018 Jan 30;9:428. doi: 10.1038/s41467-017-02650-6 (PMC5789846; doi:10.1038/s41467-017-02650-6)
Supplement: Supplementary file 7 — Supplementary Data 4 [file 41467_2017_2650_MOESM7_ESM.pdf]

Supplementary Data 4. Sequence of the plasmid containing iTol2Amp-Cryst:RFP used as a template for recombineering.

```
LOCUS       Tol2_Amp_Cryst_RFP                7724 bp ds-DNA     circular SYN 
30-AGO-2016
DEFINITION  synthetic circular DNA


REFERENCE   1  (bases 1 to 7724)
  AUTHORS   .
  TITLE     Direct Submission
  JOURNAL   Exported martes, 30 de ago de 2016 from SnapGene Viewer 3.0.3
            http://www.snapgene.com
FEATURES             Location/Qualifiers
     source          1..7724
                     /organism="synthetic DNA construct"
                     /mol_type="other DNA"
     misc_feature    683..706
                     /label=pTarBAC_HA1_iTol2_fw
                     /note="pTarBAC_HA1_iTol2_fw"
     misc_feature    complement(702..901)
                     /label=Inverted L200
                     /note="Inverted L200"
     misc_feature    complement(917..935)
                     /label=amp_HA1_control_rev
                     /note="amp_HA1_control_rev"
     misc_feature    1042..1902
                     /label=AmpR
                     /note="AmpR"
     misc_feature    1868..1887
                     /label=amp_HA2_control_fw
                     /note="amp_HA2_control_fw"
     misc_feature    1925..2074
                     /label=Inverted R150
                     /note="Inverted R150"
     primer_bind     2129..2148
                     /label=T3
                     /note="T3"
     promoter        2149..4387
                     /label=gamma Crys promoter
                     /note="gamma Crys promoter"
     CDS             4397..5107
                     /label=mCherry
                     /note="mCherry"
     polyA_signal    5149..5340
                     /label=SV40 late polyA
                     /note="SV40 late polyA"
     misc_feature    5304..5324
                     /label=cryst_HA2_control_fw
                     /note="cryst_HA2_control_fw"
     misc_feature    complement(5338..5361)
                     /label=pTarBAC_HA2_iTol2CrystRFP_rev
                     /note="pTarBAC_HA2_iTol2CrystRFP_rev"
     primer_bind     complement(5388..5407)
                     /label=T3(1)
                     /note="T3(1)"
     misc_feature    5388..5407
                     /label=ATTAACCCTCACTAAAGGGA
                     /note="ATTAACCCTCACTAAAGGGA"
     misc_feature    5410..5416
                     /label=AttB1
                     /note="AttB1"
     misc_recomb     5417..5566
                     /label=AttR1
                     /note="AttR1"
     misc_recomb     5417..5566
                     /label=AttP1
                     /note="AttP1"
```

```
     misc_recomb     5417..5430
                     /label=AttL1
                     /note="AttL1"
     misc_feature    5417..5430
                     /label=AttB1(1)
                     /note="AttB1(1)"
     misc_feature    5417..5423
                     /label=Gateway recomb1
                     /note="Gateway recomb1"
     misc_feature    5971..6981
                     /label=SpectR
                     /note="SpectR"
ORIGIN
        1 ctttcctgcg ttatcccctg attctgtgga taaccgtatt accgcctttg agtgagctga
       61 taccgctcgc cgcagccgaa cgaccgagcg cagcgagtca gtgagcgagg aagcggaaga
      121 gcgcccaata cgcaaaccgc ctctccccgc gcgttggccg attcattaat gcagctggca
      181 cgacaggttt cccgactgga aagcgggcag tgagcgcaac gcaattaata cgcgtaccgc
      241 tagccaggaa gagtttgtag aaacgcaaaa aggccatccg tcaggatggc cttctgctta
      301 gtttgatgcc tggcagttta tggcgggcgt cctgcccgcc accctccggg ccgttgcttc
      361 acaacgttca aatccgctcc cggcggattt gtcctactca ggagagcgtt caccgacaaa
      421 caacagataa aacgaaaggc ccagtcttcc gactgagcct ttcgttttat ttgatgcctg
      481 gcagttccct actctcgcgt taacgctagc atggatgttt tcccagtcac gacgttgtaa
      541 aacgacggcc agtcttaagc tcgggcccca aataatgatt ttattttgac tgatagtgac
      601 ctgttcgttg caacaaattg atgagcaatg cttttttata atgccaactt tgtacaaaaa
      661 agcaggctcc gaattcgccc ttccctgctc gagccgggcc caagtgatct ccaaaaaata
      721 agtacttttt gactgtaaat aaaattgtaa ggagtaaaaa gtactttttt ttctaaaaaa
      781 atgtaattaa gtaaaagtaa aagtattgat ttttaattgt actcaagtaa agtaaaaatc
      841 cccaaaaata atacttaagt acagtaatca agtaaaatta ctcaagtact ttacacctct
      901 ggttcttgac cccctaccac ttttcgggga aatgtgcgcg gaacccctat ttgtttattt
      961 ttctaaatac attcaaatat gtatccgctc atgagacaat aaccctgata aatgcttcaa
     1021 taatattgaa aaaggaagag tatgagtatt caacatttcc gtgtcgccct tattcccttt
     1081 tttgcggcat tttgccttcc tgtttttgct cacccagaaa cgctggtgaa agtaaaagat
     1141 gctgaagatc agttgggtgc acgagtgggt tacatcgaac tggatctcaa cagcggtaag
     1201 atccttgaga gttttcgccc cgaagaacgt tttccaatga tgagcacttt taaagttctg
     1261 ctatgtggcg cggtattatc ccgtattgac gccgggcaag agcaactcgg tcgccgcata
     1321 cactattctc agaatgactt ggttgagtac tcaccagtca cagaaaagca tcttacggat
     1381 ggcatgacag taagagaatt atgcagtgct gccataacca tgagtgataa cactgcggcc
     1441 aacttacttc tgacaacgat cggaggaccg aaggagctaa ccgctttttt gcacaacatg
     1501 ggggatcatg taactcgcct tgatcgttgg gaaccggagc tgaatgaagc cataccaaac
     1561 gacgagcgtg acaccacgat gcctgtagca atggcaacaa cgttgcgcaa actattaact
     1621 ggcgaactac ttactctagc ttcccggcaa caattaatag actggatgga ggcggataaa
     1681 gttgcaggac cacttctgcg ctcggccctt ccggctggct ggtttattgc tgataaatct
     1741 ggagccggtg agcgtgggtc tcgcggtatc attgcagcac tggggccaga tggtaagccc
     1801 tcccgtatcg tagttatcta cacgacgggg agtcaggcaa ctatggatga acgaaataga
     1861 cagatcgctg agataggtgc ctcactgatt aagcattggt aaggtaccgg catatggttc
     1921 ttgacagagg tgtaaaaagt actcaaaaat tttactcaag tgaaagtaca agtacttagg
     1981 gaaaatttta ctcaattaaa agtaaaagta tctggctaga atcttacttg agtaaaagta
     2041 aaaaagtact ccattaaaat tgtacttgag tattagatcg tggcggccgc tctagaacta
     2101 gagtcgagat ccaagcttgg ggcgcgcaat taaccctcac taaagggaac aaaagctggt
     2161 accgggcccc ccctcgagtc cataagaaag atataaataa gctggagaga atgcagaatt
     2221 gtcaactagc agtgctggaa ttggggaggg atgccatccc ctcacttttt tatttctgtg
     2281 aaatagcatc ccctcagtgt ggatgcaagt tttttgaatc ccggaactgt cttttgctcc
     2341 tcttcacttc tgcatgcaaa tacaatgcag aggtaaaata taatatagtt aaatatatag
     2401 gggattaaag gagagaaaaa attgctggga gagaagaaga aaagatagag aagtgttaaa
     2461 cagataaaga aggtggaggc atcagagagg gttatagaga aaaggagaga agaaaaaatg
     2521 aaggtattaa acagcagcag attaaggaat agtgagaaaa tgctttatat gaaataatct
     2581 tgggtaatat agaatatgat ggggatgtgc tatgttatga cacaatacac agaaaaggtg
     2641 ctttatttgc agtagctgaa ttggtagcca agaaaagtca taagcacaat ttttattttg
     2701 gggtccctat aggtcacata tgtaaatatg gttatgggag ggatgcacca ctcagagcat
     2761 ccctccactt tttttactcc aattcaagca ctgggaatga gatatgcttc attagcacct
     2821 aaacatttga caatgaccaa tgtgaatgtt agcgaccata tttgcgtcct tttgtgacca
     2881 aatggcatat gtgaacattc ctagtgtatg taataaaatt ttgcaacaaa atatttaaaa
     2941 actccagagc agatgcttaa cttagtcatg taatattcgc cagcaaatga gtatacctct
     3001 atttttaaca tcacttgtga ttttttttagt aacattccca cctttagtat ttccagattg
     3061 aaattatcat accccccccat tctttaatat agggaacact gtaccagtgg gtctgggatt
     3121 atttgtccat atattgcaat atatccaagc tgaccaactg tctcaaagtc acccctggtc
     3181 tggccaataa aacacaaaca ttctagcaga aagtaaagct ggccatagat gttgagattt
     3241 ttaaaagatc agatcctgat cgtgagacca cgatcttctc agaacgatcg tacgatcgta
```

3301 cgaatctacc atcaactaaa aagaccaatt taccaggaaa acaaagggga gctgcctgct
3361 tggccctgca aacatagaga gattgcactg gggccgacaa agattttttg acctggccga
3421 tcaatttccc gacagatgtc ggccgaaaaa tcgtaagatg tacgatcgtt cgaataccac
3481 taaccgcacg ataatttcga aggattggtc gggcttccct aaaatcggtc gttcggcaag
3541 aagaatcgtc gcgtctatgg ggaccttaag tgtacttgct ttccaaggtt aaaaattgtt
3601 acctttttat acgacactgc atggatcacc tgaaagcagg ttgggagcta caacatggaa
3661 ctagccactg ttcctttata tggttatcac tacccaattt taaataatta aactggagtt
3721 tattgatggt gtgaccacaa aattgataca gataaagaca tactgtatat attgcgttat
3781 atgacaaata cagtaacacc acattactat tttaaattat atataaaata atattaatat
3841 atctggggac ataaaatgtg gcttcttaaa cattggggtg acactgatga gattatttgg
3901 taaatgtttg atgctagcta accattgttt ggttcatttg tgtgattcaa aagtggcaaa
3961 gaatgacacc tagatgatgc caaattaagg atttactagt aaccacaatt ctttaaactg
4021 ttacaaagtg tttggaccag caactgaatt gcactgtaaa ttaccacatc agggtctccg
4081 gggtcaacgc tgctaagcaa tggactggaa aatttgtgac attgagtttt tggaataact
4141 gggaatgctg gataactctt tgggtaaagc tatggaacaa aagtaccagc atgaactaag
4201 aaatgactgt cacagcacaa catcaaacac aatgcaagac aaaggttctc tgcttttgcc
4261 ttacttgaca gaaaaagtaa gctctaggtt catcgcatgc agacagcagc aatacatgac
4321 atatataaag gctggctctt ttctcccttc cattgaactg aaacttccac tcagtcagac
4381 ttgcgggggga tccaccatgg tgagcaaggg cgaggaggat aacatggcca tcatcaagga
4441 gttcatgcgc ttcaaggtgc acatggaggg ctccgtgaac ggccacgagt tcgagatcga
4501 gggcgagggc gagggccgcc cctacgaggg cacccagacc gccaagctga aggtgaccaa
4561 gggtggcccc ctgcccttcg cctgggacat cctgtcccct cagttcatgt acggctccaa
4621 ggcctacgtg aagcaccccg ccgacatccc cgactacttg aagctgtcct tccccgaggg
4681 cttcaagtgg gagcgcgtga tgaacttcga ggacggcggc gtggtgaccg tgacccagga
4741 ctcctccctg caggacggcg agttcatcta caaggtgaag ctgcgcggca ccaacttccc
4801 ctccgacggc cccgtaatgc agaagaagac catgggctgg gaggcctcct ccgagcggat
4861 gtaccccgag gacggcgccc tgaagggcga gatcaagcag aggctgaagc tgaaggacgg
4921 cggccactac gacgctgagg tcaagaccac ctacaaggcc aagaagcccg tgcagctgcc
4981 cggcgcctac aacgtcaaca tcaagttgga catcacctcc cacaacgagg actacaccat
5041 cgtggaacag tacgaacgcg ccgagggccg ccactccacc ggcggcatgg acgagctgta
5101 caagtaactc gagcctctag aactatagtg agtcgtatta cgtagatcca gacatgataa
5161 gatacattga tgagtttgga caaaccacaa ctagaatgca gtgaaaaaaa tgctttattt
5221 gtgaaatttg tgatgctatt gctttatttg taaccattat aagctgcaat aaacaagtta
5281 acaacaacaa ttgcattcat tttatgtttc aggttcaggg ggaggtgtgg gaggtttttt
5341 aatcgatacc gtcgacctcg aggggggggcc cggtacccag cttttgttcc ctttagtgag
5401 ggttaattgc aagtttgtac aaaaaagttg aacgagaaac gtaaaatgat ataaatatca
5461 atatattaaa ttagattttg cataaaaaac agactacata atactgtaaa acacaacata
5521 tgcagtcact atgaatcaac tacttagatg gtattagtga cctgtagaat tgatctagag
5581 gatcataata agggcgaatt cgacccagct ttcttgtaca aagttggcat tataaaaaat
5641 aattgctcat caatttgttg caacgaacag gtcactatca gtcaaaataa aatcattatt
5701 tgccatccag ctgatatccc ctatagtgag tcgtattaca tggtcatagc tgtttcctgg
5761 cagctctggc ccgtgtctca aaatctctga tgttacattg cacaagataa aaatatatca
5821 tcatgcctcc tctagaccag ccaggacaga aatgcctcga cttcgctgct gcccaaggtt
5881 gccgggtgac gcacaccgtg gaaacggatg aaggcacgaa cccagtggac ataagcctgt
5941 tcggttcgta agctgtaatg caagtagcgt atgcgctcac gcaactggtc cagaaccttg
6001 accgaacgca gcggtggtaa cggcgcagtg gcggttttca tggcttgtta tgactgtttt
6061 tttggggtac agtctatgcc tcgggcatcc aagcagcaag cgcgttacgc cgtgggtcga
6121 tgtttgatgt tatggagcag caacgatgtt acgcagcagg gcagtcgccc taaaacaaag
6181 ttaaacatca tgagggaagc ggtgatcgcc gaagtatcga ctcaactatc agaggtagtt
6241 ggcgtcatcg agcgccatct cgaaccgacg ttgctggccg tacatttgta cggctccgca
6301 gtggatggcg gcctgaagcc acacagtgat attgatttgc tggttacggt gaccgtaagg
6361 cttgatgaaa caacgcggcg agctttgatc aacgaccttt tggaaacttc ggcttcccct
6421 ggagagagcg agattctccg cgctgtagaa gtcaccattg ttgtgcacga cgacatcatt
6481 ccgtggcgtt atccagctaa gcgcgaactg caatttggag aatggcagcg caatgacatt
6541 cttgcaggta tcttcgagcc agccacgatc gacattgatc tggctatctt gctgacaaaa
6601 gcaagagaac atagcgttgc cttggtaggt ccagcggcgg aggaactctt tgatccggtt
6661 cctgaacagg atctatttga ggcgctaaat gaaaccttaa cgctatggaa ctcgccgccc
6721 gactgggctg gcgatgagcg aaatgtagtg cttacgttgt cccgcatttg gtacagcgca
6781 gtaaccggca aaatcgcgcc gaaggatgtc gctgccgact gggcaatgga gcgcctgccg
6841 gcccagtatc agcccgtcat acttgaagct agacaggctt atcttggaca agaagaagat
6901 cgcttggcct cgcgcgcaga tcagttggaa gaatttgtcc actacgtgaa aggcgagatc
6961 accaaggtag tcggcaaata accctcgagc cacccatgac caaaatccct taacgtgagt
7021 tacgcgtcgt tccactgagc gtcagacccc gtagaaaaga tcaaaggatc ttcttgagat
7081 cctttttttc tgcgcgtaat ctgctgcttg caaacaaaaa aaccaccgct accagcggtg
7141 gtttgtttgc cggatcaaga gctaccaact ctttttccga aggtaactgg cttcagcaga
7201 gcgcagatac caaatactgt ccttctagtg tagccgtagt taggccacca cttcaagaac
7261 tctgtagcac cgcctacata cctcgctctg ctaatcctgt taccagtggc tgctgccagt
7321 ggcgataagt cgtgtcttac cgggttggac tcaagacgat agttaccgga taaggcgcag

```
     7381 cggtcgggct gaacgggggg ttcgtgcaca cagcccagct tggagcgaac gacctacacc
     7441 gaactgagat acctacagcg tgagcattga gaaagcgcca cgcttcccga agggagaaag
     7501 gcggacaggt atccggtaag cggcagggtc ggaacaggag agcgcacgag ggagcttcca
     7561 gggggaaacg cctggtatct ttatagtcct gtcgggtttc gccacctctg acttgagcgt
     7621 cgatttttgt gatgctcgtc aggggggcgg agcctatgga aaaacgccag caacgcggcc
     7681 tttttacggt tcctggcctt ttgctggcct tttgctcaca tgtt
//
```
