# Supplementary material for: Tbx5a lineage tracing shows cardiomyocyte plasticity during zebrafish heart regeneration
Source: Nat Commun. 2018 Jan 30;9:428. doi: 10.1038/s41467-017-02650-6 (PMC5789846; doi:10.1038/s41467-017-02650-6)
Supplement: Supplementary file 8 — Supplementary Data 5 [file 41467_2017_2650_MOESM8_ESM.pdf]

Supplementary Data 5. Sequence of the plasmid containing loxP_tagBFP_loxP_mCherry-NTR- flp-kan-flp used as a template for recombineering.

```
LOCUS       loxP-myctagBFP-STOP-lox-mCherryNTR fl Kan               7515 bp
ds-DNA     circular SYN 30-AGO-2016
DEFINITION  synthetic circular DNA

REFERENCE   1  (bases 1 to 7515)
  AUTHORS   .
  TITLE     Direct Submission
  JOURNAL   Exported martes, 30 de ago de 2016 from SnapGene Viewer 3.0.3
            http://www.snapgene.com
FEATURES             Location/Qualifiers
     source          1..7515
                     /organism="synthetic DNA construct"
                     /mol_type="other DNA"
     misc_feature    432..465
                     /label=LoxP
                     /note="LoxP"
     misc_feature    466..472
                     /label=kozak
                     /note="kozak"
     misc_feature    473..475
                     /label=ATG
                     /note="ATG"
     misc_feature    476..505
                     /label=myc
                     /note="myc"
     misc_feature    506..1225
                     /label=tagBFP
                     /note="tagBFP"
     misc_feature    1226..1729
                     /label=Rabbit beta globin terminator
                     /note="Rabbit beta globin terminator"
     misc_feature    1730..1763
                     /label=LoxP(1)
                     /note="LoxP(1)"
     misc_feature    1778..1784
                     /label=kozak(1)
                     /note="kozak(1)"
     misc_feature    1785..2493
                     /label=mCherry
                     /note="mCherry"
     misc_feature    2494..2543
                     /label=Linker
                     /note="Linker"
     misc_feature    2544..3197
                     /label=NTR
                     /note="NTR"
     misc_feature    3216..3454
                     /label=SV40 poliA
                     /note="SV40 poliA"
     misc_feature    3491..3525
                     /label=FRT
                     /note="FRT"
     misc_feature    3780..3918
                     /label=Sv40 poliA
                     /note="Sv40 poliA"
     misc_feature    3969..4761
                     /label=KanR
                     /note="KanR"
     misc_feature    4842..4876
                     /label=FRT(1)
                     /note="FRT(1)"
ORIGIN
        1 tcgcgcgttt cggtgatgac ggtgaaaacc tctgacacat gcagctcccg gagacggtca
       61 cagcttgtct gtaagcggat gccgggagca gacaagcccg tcagggcgcg tcagcgggtg
      121 ttggcgggtg tcggggctgg cttaactatg cggcatcaga gcagattgta ctgagagtgc
```

```
 181 accatatgcg gtgtgaaata ccgcacagat gcgtaaggag aaaataccgc atcaggcgcc
 241 attcgccatt caggctgcgc aactgttggg aagggcgatc ggtgcgggcc tcttcgctat
 301 tacgccagct ggcgaaaggg ggatgtgctg caaggcgatt aagttgggta acgccagggt
 361 tttcccagtc acgacgttgt aaaacgacgg ccagtgccaa gctcggcgcg cccgggaccg
 421 gtggatccac tataacttcg tatagcatac attatacgaa gttatcgcca ccatggagca
 481 gaagctgatc tcagaggagg acctgatgag cgagctgatt aaggagaaca tgcacatgaa
 541 gctgtacatg gagggcaccg tggacaacca tcacttcaag tgcacatccg agggcgaagg
 601 caagccctac gagggcaccc agaccatgag aatcaaggtg gtcgagggcg gccctctccc
 661 cttcgccttc gacatcctgg ctactagctt cctctacggc agcaagacct tcatcaacca
 721 cacccagggc atccccgact tcttcaagca gtccttccct gagggcttca catgggagag
 781 agtcaccaca tacgaagacg gggcgtgct gaccgctacc caggacacca gcctccagga
 841 cggctgcctc atctacaacg tcaagatcag aggggtgaac ttcacatcca acggccctgt
 901 gatgcagaag aaaacactcg gctgggaggc cttcaccgag acgctgtacc ccgctgacgg
 961 cggcctggaa ggcagaaacg acatggccct gaagctcgtg ggcgggagcc atctgatcgc
1021 aaacatcaag accacatata gatccaagaa acccgctaag aacctcaaga tgcctggcgt
1081 ctactatgtg gactacagac tggaaagaat caaggaggcc aacaacgaga cctacgtcga
1141 gcagcacgag gtggcagtgg ccagatactg cgacctccct agcaaactgg ggcacaagct
1201 taattaaagc ggccgcactc ctcaggtgca ggctgcctat cagaaggtgg tggctggtgt
1261 ggccaatgcc ctggctcaca aataccactg agatcttttt ccctctgcca aaaattatgg
1321 ggacatcatg aagccccttg agcatctgac ttctggctaa taaaggaaat ttattttcat
1381 tgcaatagtg tgttggaatt ttttgtgtct ctcactcgga aggacatatg ggagggcaaa
1441 tcatttaaaa catcagaatg agtatttggt ttagagtttg gcaacatatg cccatatgct
1501 ggctgccatg aacaaaggtt ggctataaag aggtcatcag tatatgaaac agccccctgc
1561 tgtccattcc ttattccata gaaaagcctt gacttgaggt tagatttttt ttatattttg
1621 ttttgtgtta tttttttctt taacatccct aaaattttcc ttacatgttt tactagccag
1681 atttttcctc ctctcctgac tactcccagt catagctgtc cctcttctca taacttcgta
1741 tagcatacat tatacgaagt tatcggatcc catcgatcgc caccatggtg agcaagggcg
1801 aggaggataa catggccatc atcaaggagt tcatgcgctt caaggtgcac atggagggct
1861 ccgtgaacgg ccacgagttc gagatcgagg gcgagggcga gggccgcccc tacgagggca
1921 cccagaccgc caagctgaag gtgaccaagg gtggccccct gcccttcgcc tgggacatcc
1981 tgtcccctca gttcatgtac ggctccaagg cctacgtgaa gcaccccgcc gacatccccg
2041 actacttgaa gctgtccttc cccgagggct tcaagtggga gcgcgtgatg aacttcgagg
2101 acggcggcgt ggtgaccgtg acccaggact cctccctgca ggacggcgag ttcatctaca
2161 aggtgaagct gcgcggcacc aacttcccct ccgacggccc cgtaatgcag aagaagacca
2221 tgggctggga ggcctcctcc gagcggatgt accccgagga cggcgccctg aagggcgaga
2281 tcaagcagag gctgaagctg aaggacggcg gccactacga cgctgaggtc aagaccacct
2341 acaaggccaa gaagcccgtg cagctgcccg gcgcctacaa cgtcaacatc aagttggaca
2401 tcacctccca caacgaggac tacaccatcg tggaacagta cgaacgcgcc gagggccgcc
2461 actccaccgg cggcatggac gagctgtaca agtccggact cagatctcga gctcaagctt
2521 cgaattcagg cggcggcgcc gccatggaca tcatctctgt ggctctgaag agacatagca
2581 caaaggcttt tgatgccagc aagaaactga cccctgagca ggctgagcag atcaagacac
2641 tgctgcagta cagcccaagc agccagaaca gccagccttg gcattttatt gtggcttcta
2701 cagaggaggg aaaggctaga gtggctaagt ctgctgctgg aaattatgtg ttctctgaga
2761 gaaagatgct ggatgcctct catgtggtgg tgttctgtgc taagacagcc atggatgatg
2821 tgtggctgaa actggtggtg gaccaggagg atgctgatgg aagatttgcc acacctgagg
2881 ctaaggctgc taatgataag ggaagaaagt tcacagctga tatgcacaga aaggacctgc
2941 atgatgatgc tgagtggatg gctaagcagg tgtatctgaa tgtgggaaac ttcctgctgg
3001 gagtggctgc tctgggactg gatgctgtgc ctattgaggg atttgatgct gctatcctgg
3061 atgctgagtt tggactgaag gagaagggat acaccagcct ggtggtggtg ccagtgggac
3121 accactctgt ggaggatttt aatgctacac tgcctaagag cagactgcct cagaacatca
3181 ccctgacaga ggtgtaagag ctcgaattcg cccttaggcc tgcaggatca taatcagcca
3241 taccacattt gtagaggttt tacttgcttt aaaaaacctc ccacacctcc ccctgaacct
3301 gaaacataaa atgaatgcaa ttgttgttgt taacttgttt attgcagctt ataatggtta
3361 caaataaagc aatagcatca caaatttcac aaataaagca tttttttcac tgcattctag
3421 ttgtggtttg tccaaactca tcaatgtatc ttaaactagg ggggatctgc tagcagataa
3481 ctgatcagct tgaagttcct atactttcta gagaatagga acttcggaat aggaacttca
3541 agatccccct ggcgaaaggg ggatgtgctg caaggcgatt aagttgggta acgccagggt
3601 tttcgtcagg tggcactttt cggggaaatg tgcgcggaac ccctatttgt ttatttttct
3661 aaatacattc aaatatgtat ccgctcatga gacaataacc ctgataaatg cttcaataat
3721 attgaaaaag gaagagtcct gaggcggaaa gaaccagctg tggaatgtgt gtcagttagg
3781 gtgtggaaag tccccaggct ccccagcagg cagaagtatg caaagcatgc atctcaatta
3841 gtcagcaacc aggtgtggaa agtccccagg ctccccagca ggcagaagta tgcaaagcat
3901 gcatctcaat tagtcagtaa ccatagtccg caaagatcga tcaagagaca ggatgaggat
3961 cgtttcgcat gattgaacaa gatggattgc acgcaggttc tccggccgct tgggtggaga
4021 ggctattcgg ctatgactgg gcacaacaga caatcggctg ctctgatgcc gccgtgttcc
4081 ggctgtcagc gcaggggcgc ccggttcttt ttgtcaagac cgacctgtcc ggtgccctga
4141 atgaactgca agacgaggca gcgcggctat cgtggctggc cacgacgggc gttccttgcg
4201 cagctgtgct cgacgttgtc actgaagcgg gaagggactg gctgctattg ggcgaagtgc
```

```
4261 cggggcagga tctcctgtca tctcaccttg ctcctgccga gaaagtatcc atcatggctg
4321 atgcaatgcg gcggctgcat acgcttgatc cggctacctg cccattcgac caccaagcga
4381 aacatcgcat cgagcgagca cgtactcgga tggaagccgg tcttgtcgat caggatgatc
4441 tggacgaaga gcatcagggg ctcgcgccag ccgaactgtt cgccaggctc aaggcgagca
4501 tgcccgacgg cgaggatctc gtcgtgaccc atggcgatgc ctgcttgccg aatatcatgg
4561 tggaaaatgg ccgcttttct ggattcatcg actgtggccg gctgggtgtg gcggaccgct
4621 atcaggacat agcgttggct acccgtgata ttgctgaaga gcttggcggc gaatgggctg
4681 accgcttcct cgtgctttac ggtatcgccg ctcccgattc gcagcgcatc gccttctatc
4741 gccttcttga cgagttcttc tgaggggatc ggcaataaaa agacagaata aaacgcacgg
4801 gtgttgggtc gtttgttcgg atccgagctt caaaagcgct ctgaagttcc tatactttct
4861 agagaatagg aacttcggaa tagtaacttc tccatggtag cctccaaaaa agcctcctca
4921 ctacttctgg actagtgcgg ccgcgcgttt aagtggcctg tgactacgca gtgtcattat
4981 cttacagaat aggtttggac ccagatgttg tggtgcctgg aggttttaaa ttttggaaag
5041 cccatctcag gaaaaaaaaa aaccacaaaa ttatgaatac agaggtgttg ggctgcccct
5101 gggacttgaa agggagcttg taatttgggg accagaggtt taaatcccta tagtttcact
5161 gtgaattatt caccttgctc aaactttcac tctgagaggc tgcctgatta ggtgcctagc
5221 gagaccagct ggatgtagct ttgtgctctt tcctctgttg attttctccg cttaattaag
5281 aattcgtaat catgtcatag ctgtttcctg tgtgaaattg ttatccgctc acaattccac
5341 acaacatacg agccggaagc ataaagtgta aagcctgggg tgcctaatga gtgagctaac
5401 tcacattaat tgcgttgcgc tcactgcccg ctttccagtc gggaaacctg tcgtgccagc
5461 tgcattaatg aatcggccaa cgcgcgggga gaggcggttt gcgtattggg cgctcttccg
5521 cttcctcgct cactgactcg ctgcgctcgg tcgttcggct gcggcgagcg gtatcagctc
5581 actcaaaggc ggtaatacgg ttatccacag aatcagggga taacgcagga aagaacatgt
5641 gagcaaaagg ccagcaaaag gccaggaacc gtaaaaaggc cgcgttgctg gcgtttttcc
5701 ataggctccg cccccctgac gagcatcaca aaaatcgacg ctcaagtcag aggtggcgaa
5761 acccgacagg actataaaga taccaggcgt ttccccctgg aagctccctc gtgcgctctc
5821 ctgttccgac cctgccgctt accggatacc tgtccgcctt tctcccttcg ggaagcgtgg
5881 cgctttctca aagctcacgc tgtaggtatc tcagttcggt gtaggtcgtt cgctccaagc
5941 tgggctgtgt gcacgaaccc cccgttcagc ccgaccgctg cgccttatcc ggtaactatc
6001 gtcttgagtc caacccggta agacacgact tatcgccact ggcagcagcc actggtaaca
6061 ggattagcag agcgaggtat gtaggcggtg ctacagagtt cttgaagtgg tggcctaact
6121 acggctacac tagaagaaca gtatttggta tctgcgctct gctgaagcca gttaccttcg
6181 gaaaaagagt tggtagctct tgatccggca aacaaaccac cgctggtagc ggtggttttt
6241 ttgtttgcaa gcagcagatt acgcgcagaa aaaaaggatc tcaagaagat cctttgatct
6301 tttctacggg gtctgacgct cagtggaacg aaaactcacg ttaagggatt ttggtcatga
6361 gattatcaaa aaggatcttc acctagatcc ttttaaatta aaaatgaagt tttaaatcaa
6421 tctaaagtat atatgagtaa acttggtctg acagttacca atgcttaatc agtgaggcac
6481 ctatctcagc gatctgtcta tttcgttcat ccatagttgc ctgactcccc gtcgtgtaga
6541 taactacgat acgggagggc ttaccatctg gccccagtgc tgcaatgata ccgcgagacc
6601 cacgctcacc ggctccagat ttatcagcaa taaaccagcc agccggaagg gccgagcgca
6661 gaagtggtcc tgcaacttta tccgcctcca tccagtctat taattgttgc cgggaagcta
6721 gagtaagtag ttcgccagtt aatagtttgc gcaacgttgt tgccattgct acaggcatcg
6781 tggtgtcacg ctcgtcgttt ggtatggctt cattcagctc cggttcccaa cgatcaaggc
6841 gagttacatg atcccccatg ttgtgcaaaa aagcggttag ctccttcggt cctccgatcg
6901 ttgtcagaag taagttggcc gcagtgttat cactcatggt tatggcagca ctgcataatt
6961 ctcttactgt catgccatcc gtaagatgct tttctgtgac tggtgagtac tcaaccaagt
7021 cattctgaga atagtgtatg cggcgaccga gttgctcttg cccggcgtca atacgggata
7081 ataccgcgcc acatagcaga actttaaaag tgctcatcat tggaaaacgt tcttcggggc
7141 gaaaactctc aaggatctta ccgctgttga gatccagttc gatgtaaccc actcgtgcac
7201 ccaactgatc ttcagcatct tttactttca ccagcgtttc tgggtgagca aaaacaggaa
7261 ggcaaaatgc cgcaaaaaag ggaataaggg cgacacggaa atgttgaata ctcatactct
7321 tcctttttca atattattga agcatttatc agggttattg tctcatgagc ggatacatat
7381 ttgaatgtat ttagaaaaat aaacaaatag gggttccgcg cacatttccc cgaaaagtgc
7441 cacctgacgt ctaagaaacc attattatca tgacattaac ctataaaaat aggcgtatca
7501 cgaggccctt tcgtc
//
```
